# CAS directly interacts with vinculin to control mechanosensing and focal adhesion dynamics

**DOI:** 10.1007/s00018-013-1450-x

**Published:** 2013-08-25

**Authors:** Radoslav Janoštiak, Jan Brábek, Vera Auernheimer, Zuzana Tatárová, Lena A. Lautscham, Tuli Dey, Jakub Gemperle, Rudolf Merkel, Wolfgang H. Goldmann, Ben Fabry, Daniel Rösel

**Affiliations:** 1 Department of Cell Biology, Faculty of Science, Charles University in Prague, Czech Republic, Vinicna 7, 128 43 Prague, Czech Republic; 2 Biophysics Group, Department of Physics, University of Erlangen-Nuremberg, Erlangen, Germany; 3 Forschungszentrum Jülich GmbH, ICS-7, Biomechanics, Jülich, Germany

**Keywords:** CAS, Focal adhesions, Mechanosensing, Vinculin, Src, Traction forces

## Abstract

Focal adhesions are cellular structures through which both mechanical forces and regulatory signals are transmitted. Two focal adhesion-associated proteins, Crk-associated substrate (CAS) and vinculin, were both independently shown to be crucial for the ability of cells to transmit mechanical forces and to regulate cytoskeletal tension. Here, we identify a novel, direct binding interaction between CAS and vinculin. This interaction is mediated by the CAS SRC homology 3 domain and a proline-rich sequence in the hinge region of vinculin. We show that CAS localization in focal adhesions is partially dependent on vinculin, and that CAS–vinculin coupling is required for stretch-induced activation of CAS at the Y410 phosphorylation site. Moreover, CAS–vinculin binding significantly affects the dynamics of CAS and vinculin within focal adhesions as well as the size of focal adhesions. Finally, disruption of CAS binding to vinculin reduces cell stiffness and traction force generation. Taken together, these findings strongly implicate a crucial role of CAS–vinculin interaction in mechanosensing and focal adhesion dynamics.

**Electronic supplementary material:**

The online version of this article (doi:10.1007/s00018-013-1450-x) contains supplementary material, which is available to authorized users.

## Introduction

Crk-associated substrate (CAS) is an important substrate of Src. CAS is dominantly localized in focal adhesions of adherent cells and plays a central role in the integrin-mediated control of cell behavior [1]. The re-expression of CAS in *cas*-deficient mouse embryonic fibroblasts (MEFs) altered by oncogenic Src was shown to elevate cell invasiveness [2] and lung metastasis formation [3]. BCAR1 (breast cancer anti-estrogen resistance), the human equivalent of CAS, was first discovered in a screen for anti-estrogen drug-resistant genes in breast cancer cells [4]. Increased CAS/BCAR1 levels in breast cancer patients are associated with premature disease recurrence, decreased response to tamoxifen treatment, and lower survival rate [5].

The structure of CAS consists of an N-terminal SRC homology 3 (SH3) domain, a C-terminally localized Src-binding domain (SBD), and a Cas-family C-terminal homology (CCH) domain. The CAS central region comprises of a substrate domain (SD), which is characterized by 15 Tyr- X- X- Pro (YxxP) motifs. Src family kinases seem to phosphorylate numerous, if not all, of the CAS SD YxxP tyrosines either by binding directly to the CAS SBD, or by indirect association with CAS via a FAK bridge [6, 7]. CAS SD tyrosine phosphorylation, in untransformed cells, takes place at the integrin-mediated adhesion sites [8], and has been shown to be associated with integrin signaling pathways that regulate cell movement and survival [9–12].

The SH3 domain of CAS is known to interact with polyproline motifs on numerous proteins [13], which include FAK and PYK2/RAFTK kinases [14], FRNK [15], PTP–PEST [16], C3G [17], PTP1B [18], CMS [19], and CIZ [20]. The interaction between the prominent focal adhesion protein, FAK, and the CAS SH3 domain [13, 15, 21] contributes to the phosphorylation of SD tyrosine and is further enhanced by Src that is bound to the FAK autophosphorylation site [6]. Accordingly, CAS SH3 domain deletion diminishes the phosphorylation of CAS SD tyrosine [6] and greatly reduces the localization of CAS to focal adhesions [22]. Detection of CAS in focal adhesions of FAK null cells [23], however, also indicates the presence of other SH3 domain binding partners.

CAS acts as a primary force sensor, transducing forces into mechanical extension of the substrate domain and thereby priming its phosphorylation and subsequent activation of downstream signaling [24]. Stretch-dependent tyrosine phosphorylation of CAS by Src family kinases is involved in force-dependent activation of the MAP kinase cascade and the small GTPase Rap1 [25, 26]. Details of how mechanical forces are coupled to the CAS protein are currently elusive. It has been suggested that mechanical stress leads to force-dependent extension of the CAS substrate domain and enhances its susceptibility to phosphorylation [24].

In this study, we identified a novel direct interaction of CAS with vinculin mediated by the CAS SH3 domain and a proline-rich sequence in the hinge region of vinculin. We found that this interaction is important for CAS localization in focal adhesions, focal adhesion dynamics, mechanosensing, and traction force generation.

## Results

### CAS SH3 domain interacts with the polyproline motif PPKP on vinculin

**Fig. 1:**
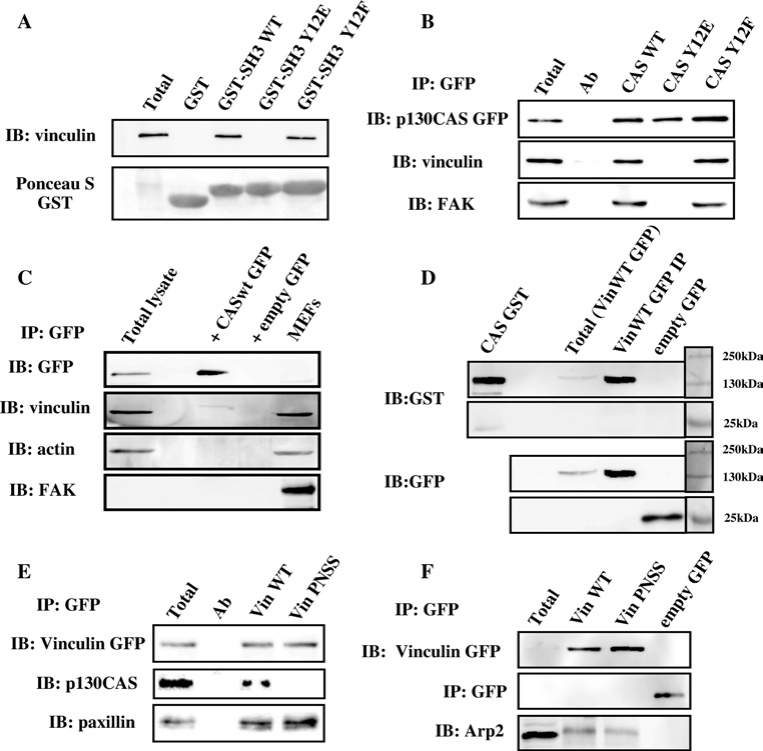
The CAS SH3 domain interacts with the polyproline region of vinculin. **a** Binding of vinculin to SH3 domains of CAS WT, CAS Y12E, and CAS Y12F fused with GST was analyzed with pull-down assays by immunoblotting. Vinculin was detected with an anti vinculin antibody. GST fused SH3 domains were detected by Ponceau S staining. Aliquots of total cell lysates (total) were used as a control. **b** GFP CAS was immunoprecipitated from CAS−/− MEFs expressing CAS Y12 variants, and binding of vinculin and FAK (as a control) was analyzed using vinculin and FAK antibodies. **c** GFP CAS WT was immunoprecipitated from FAK−/− MEFs and binding of vinculin was analyzed using vinculin antibody. **d** In a far-Western experiment, Vin WT GFP or GFP immunoprecipitated from Vin−/− MEFs expressing the GFP constructs were transferred to nitrocellulose membranes and incubated with recombinant CAS–GST, followed by detection with anti-GST antibody. Loading controls of GFP constructs were analyzed by anti-GFP antibody. As a positive control for anti-GST reactivity, purified CAS–GST was run alongside. **e** GFP vinculin was immunoprecipitated from Vin−/− MEFs re-expressing GFP-fused Vin WT or Vin PNSS (PKPP sequence in the proline-rich region changed to PNSS), and binding of CAS and paxillin was detected with CAS and paxillin antibodies. **f** GFP vinculin was immunoprecipitated from Vin−/− MEFs re-expressing GFP-fused Vin WT or Vin PNSS (PKPP sequence in proline-rich region changed to PNSS), and binding of Arp2 was detected with Arp2 antibody

The importance of CAS SH3 domain in CAS-mediated signaling is well established. The SH3 domain targets CAS into focal adhesions and significantly contributes to CAS substrate domain phosphorylation and downstream signaling through the CAS/Crk scaffold [8, 22, 23]. We have previously shown that phosphorylation on tyrosine 12 within the SH3 domain of CAS suppresses FAK binding and deactivates CAS-mediated signaling [27, 28]. To identify novel interacting partners that can differentially bind to a non-phosphorylated CAS SH3 domain, we employed peptide mass fingerprinting of proteins differentially pulled-down by a GST fused CAS SH3 domain from HeLa cell lysates. Apart from known interaction partners of the CAS SH3 domain such as FAK and CMS, we found vinculin as a novel potential binding partner of CAS. CAS–vinculin interaction was confirmed to be specific for non-phosphorylated CAS SH3 domain using a GST pull-down assay with immunoblot analysis (Fig. 1a). Vinculin binding was only present in CAS–SH3 domain constructs with WT tyrosine at position 12 or with a non-phosphorylatable mutation Y12F (Tyr 12 changed to phenylalanine). No vinculin binding was observed in the case of a mutation Y12E (Tyr 12 changed to glutamic acid). The Y12E was previously shown to also abolish specific binding of other CAS–SH3 interacting partners, similarly to Tyr 12 phosphorylation [27]. Even though the Y12E mutation may not truly mimic the structure of phosphorylated Tyr 12, it was clearly shown that substitution of Tyr 12 and analogous Tyr in SH3 domains to Glu in its effect on binding capacity of the SH3 domains mimics phosphorylation [27, 28]. We will thus further refer to this mutation as a phosphomimicking. Results from GST-pull down were further confirmed by immuno-precipitation of full-length CAS tagged with GFP (Fig. 1b).

As the CAS SH3 domain is also known to interact with FAK [13], we investigated whether CAS SH3 interaction with vinculin is independent of FAK. GFP–CAS was immuno-precipitated from FAK−/− cells and Western-blot analysis showed that CAS also interacts with vinculin in FAK-deficient cells (Fig. 1c). Moreover, GST-pull-down assays with the WT CAS SH3 domain were performed with or without FAK depletion using an anti-FAK antibody. FAK depletion in cell lysate did not affect the interaction of WT CAS SH3 domain and vinculin (Fig.S1), suggesting that the CAS–vinculin interaction is FAK independent and potentially direct. In a far-Western experiment, we confirmed that CAS directly interacts with vinculin (Fig. 1d).

We then searched for the binding motif on vinculin that is responsible for CAS SH3 domain binding. We analyzed CAS SH3 binding motifs among known CAS SH3 binding partners and identified as a conserved motif a PXKP sequence (Table S1). In vinculin, the P860PKP sequence in the proline-rich region of vinculin, also known as hinge region [29], is a single potential CAS SH3 binding motif. To abolish the capability of CAS SH3 domain binding to vinculin, the in silico-identified SH3 domain-binding motif **PPKP**P was mutated into **PPNS**S (PNSS). The importance of the PPKP motif for vinculin-CAS interaction was verified by co-immunoprecipitation experiments with full-length CAS and vinculin PNSS mutant or vinculin WT. Binding of CAS to vinculin carrying the PNSS mutation was abolished (Fig. 1e). In contrast, the binding of paxillin and Arp2, which bind the tail region of vinculin [30] and proline-rich region surrounding P776 and P878 [31], respectively, was not significantly affected by this mutation (Fig. 1e, f). This further confirmed that CAS–vinculin interaction is through direct binding, as a potential linker molecule would need to recognize the **PPKP**P binding motif on vinculin and at the same time would need to bind to the CAS-SH3 domain, and both with higher affinity than a direct CAS–vinculin interaction, which is highly unlikely.

The interaction of CAS with vinculin in living cells was assessed using fluorescent confocal microscopy. CAS−/− cells were transfected with CAS Y12 variants that were N-terminally tagged with GFP, and the co-localization with vinculin was examined. As expected, CAS WT or Y12F CAS (both interact with vinculin, see Fig. 1b) were found to be co-localized with vinculin. CAS with a phosphomimicking mutation Y12E, that blocks interaction with vinculin, did not co-localize with vinculin in focal adhesions (Fig. S2).

Taken together, these results suggest that CAS directly interacts with vinculin, and that the CAS–vinculin interaction can be potentially regulated by phosphorylation of Tyr12 on the SH3 domain of CAS [27].

### CAS localization in focal adhesion is partially dependent on FAK, but also on vinculin

**Fig. 2:**
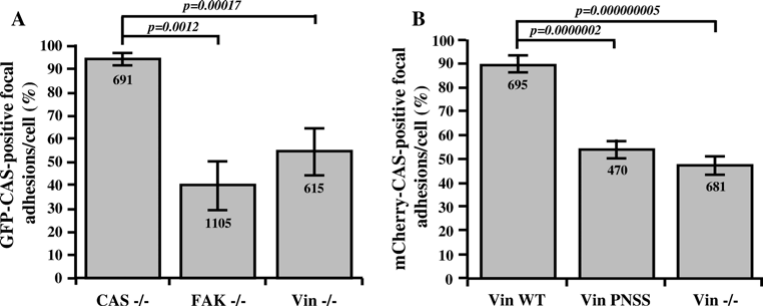
CAS localization in focal adhesions is dependent on FAK and vinculin. The *bar graphs* show the percentage of focal adhesions stained positive for GFP–CAS (**a**) or for mCherry-CAS (**b**). Focal adhesions were considered CAS-positive if the GFP–CAS or mCherry-CAS signal is at least double the signal in cytoplasm, adjacent to the focal adhesions, indicated by paxillin staining.* Numbers in columns* indicate number of analyzed focal adhesions and *error bars* represent standard deviation

FAK and vinculin belong to fundamental components of the focal adhesions [32], both interact with CAS, and thus both can be responsible for CAS targeting to focal adhesions. To differentially evaluate the importance of CAS binding to FAK versus vinculin, we tested the dependence of CAS focal adhesion targeting on these two proteins. Full-length GFP–CAS WT was expressed in CAS−/−, FAK−/−, and vinculin−/− cells, and its localization to focal adhesions was assessed by fluorescence confocal microscopy. Localization of GFP–CAS to focal adhesions was examined by differential co-localization with paxillin. Focal adhesions were identified as elongated structures with localization of paxillin and F-actin (Fig. S3A). In CAS−/− cells re-expressing GFP–CAS, CAS was present in nearly all focal adhesions. In FAK−/− cells, GFP–CAS was found only in 40 % of focal adhesions, and in Vin−/− cells, GFP–CAS was found in 54 % of focal adhesions (Fig. 2a). Re-expression of vinculin WT in Vin−/− cells, but not vinculin carrying the PNSS mutation, restored the localization of CAS in focal adhesions (Fig. 2b, Fig. S3B). These results suggest that both FAK and vinculin are responsible for focal adhesion targeting of CAS.

### CAS–vinculin interaction is important for focal adhesion dynamics

**Fig. 3:**
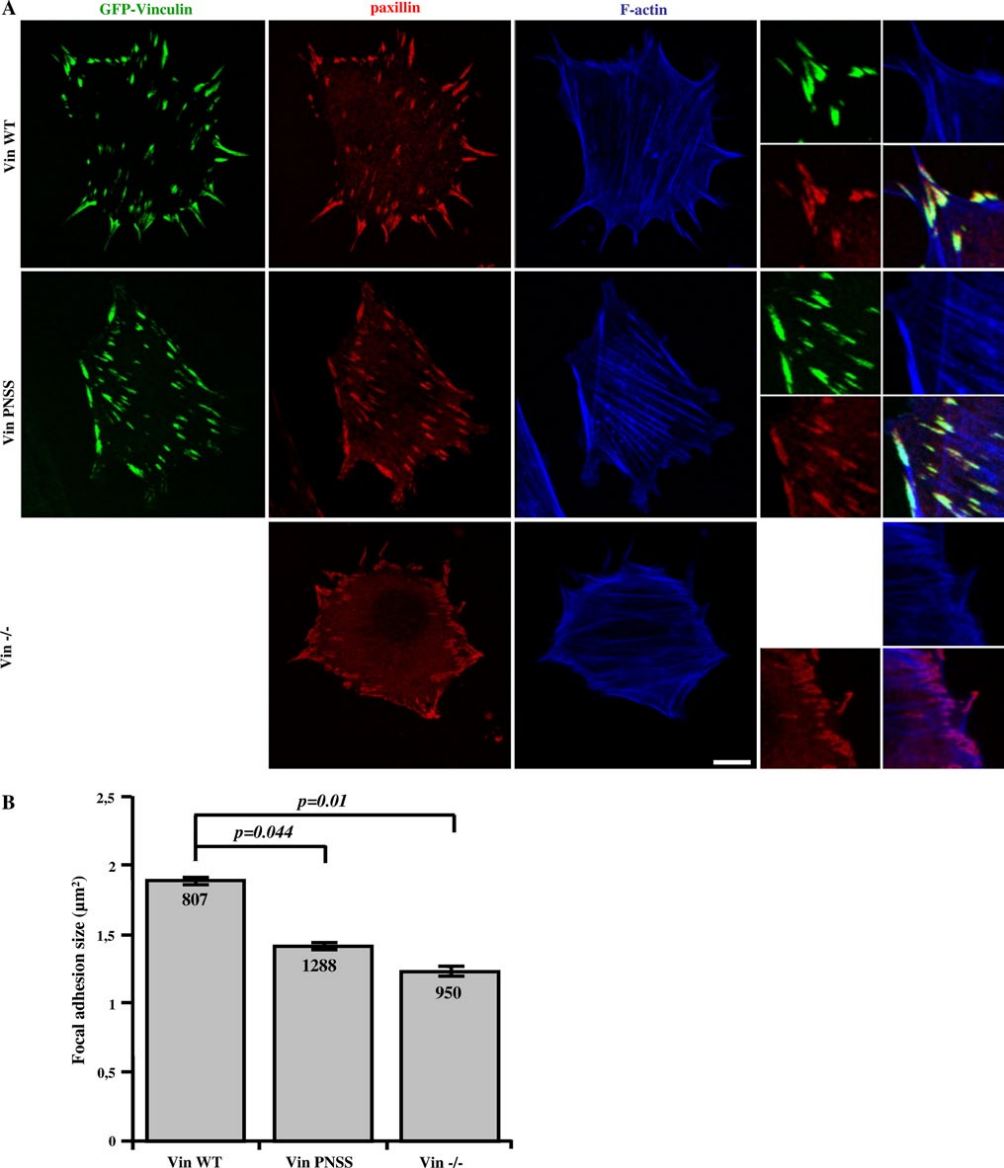
CAS–vinculin interaction affects focal adhesion size. **a** Vin−/− MEFs re-expressing either Vin WT or Vin PNSS C-terminally fused with GFP were grown on fibronectin-coated coverslips and stained for paxillin (focal adhesion marker) and F-actin. Focal adhesion size was determined using ImageJ software. *Scale bar* 10 μm (**b**). The *histogram bars* represent average size of adhesion structures in cells deficient in vinculin, or re-expressing either Vin WT or Vin PNSS mutant.* Numbers in columns* indicate number of analyzed focal adhesions

We have previously shown that phosphorylation of tyrosine 12 within the CAS SH3 domain is important for the dynamics of focal adhesions [27]. Focal adhesions are relatively stable structures, but a continuous exchange of proteins takes place. To explore the influence of CAS–vinculin interaction on focal adhesions, we first analyzed their mean size using fluorescence microscopy (Fig. 3a). The size of focal adhesions in cells expressing WT vinculin [33] was nearly 2 μm^2^, whereas the size of focal adhesions in cells lacking vinculin or in cells expressing the mutated PNSS vinculin (Vin PNSS) was significantly smaller (Vin−/−: 1,2 μm^2^, VinPNSS: 1,4 μm^2^) (Fig. 3b), suggesting that CAS targeting to vinculin is important for the formation of focal adhesions.

**Fig. 4:**
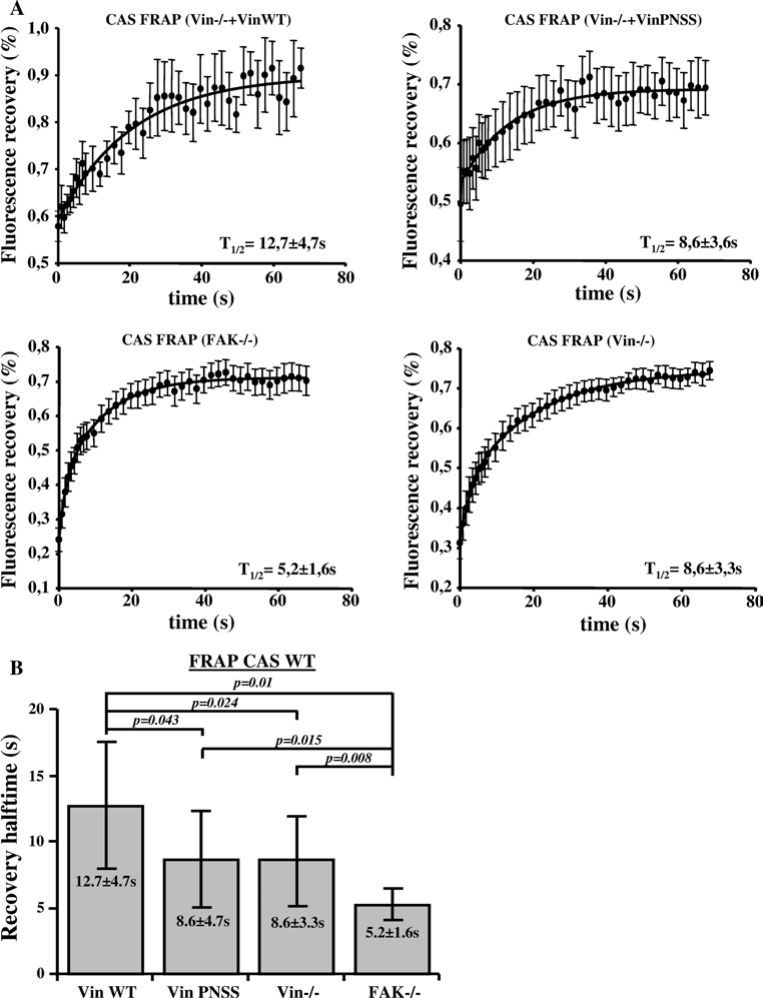
Dependence of CAS dynamics on FAK and vinculin within focal adhesions. **a** FRAP curves of CAS-Venus associated with focal adhesions in MEFs lacking FAK or vinculin, or re-expressing either Vin WT or mutated Vin PNSS. *Numbers* indicate average half-maximum recovery times (*t*1/2). **b** The *bar* plot shows average half-maximum recovery times of CAS-Venus in different MEFs. *Error bars* represent standard errors

Changes in the inner dynamics of focal adhesion proteins are linked to changes in focal adhesions assembly and disassembly rates [34, 35] and maturation [36, 37]. Therefore, we investigated the influence of CAS–vinculin interaction on the exchange dynamics of CAS within focal adhesions using the FRAP technique. The exchange dynamics of CAS in focal adhesions was measured in FAK−/− cells, Vin−/− cells, and Vin−/− cells re-expressing either Vin WT or Vin PNSS mutants. The half-maximum recovery time after photobleaching (*t*1/2) of CAS fused with a Venus variant of GFP was quantified (Fig. 4a) [22]. CAS exchange dynamics in Vin−/− cells (*t*1/2 = 8.6 ± 3.3 s) and cells re-expressing a Vin PNSS mutant (*t*1/2 = 8.6 ± 3.6 s) was significantly faster compared to cells re-expressing Vin WT (*t*1/2 = 12.7 ± 4.7 s) (Fig. 4b). CAS exchange dynamics in FAK−/− cells was also greatly accelerated (*t*1/2 = 5.2 ± 1.6 s) (Fig. 4b). These results indicate that both binding of CAS to FAK and to vinculin slow down CAS exchange dynamics in focal adhesions.

**Fig. 5:**
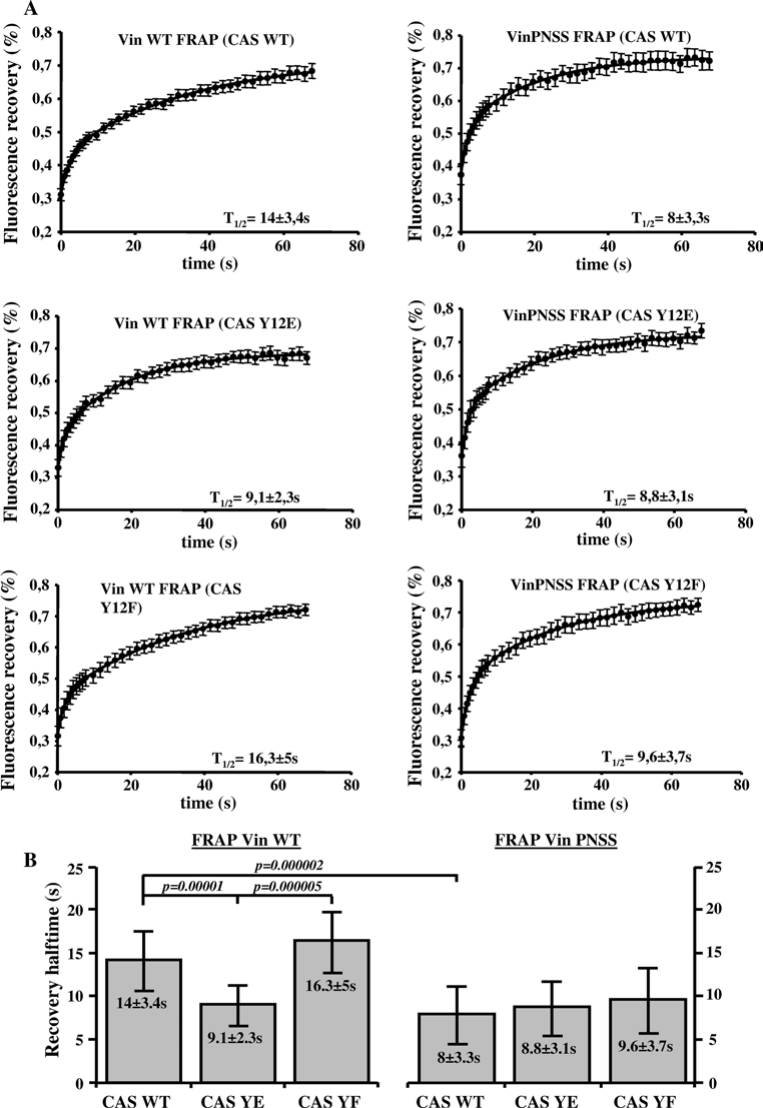
Dependence of vinculin dynamics on CAS–vinculin interaction. **a** FRAP curves of GFP-vinculin WT (*left side*) or GFP-vinculin PNSS (*right side*) associated with focal adhesions in CAS−/− MEFs re-expressing indicated CAS variants. *Numbers* in plot indicate average half-maximum recovery times (*t*1/2). **b** The* bar plot* shows half-maximum recovery times of GFP-fused Vin WT (*left*) and GFP-vinculin PNSS (*right*) in CAS−/− MEFs re-expressing indicated CAS variants. *Error bars* represent standard errors

To confirm these results, we tested whether efficient CAS–vinculin binding also slows down the vinculin exchange dynamics in focal adhesions. CAS−/− cells re-expressing the CAS WT or CAS tyrosine 12 variants [27] were transfected to express GFP tagged Vin WT. FRAP experiments revealed a similar, slow dynamics of vinculin in cells expressing vinculin binding proficient CAS Y12F (*t*1/2 = 16.3 ± 5 s) and CAS WT (*t*1/2 = 14 ± 3.4 s). However, vinculin dynamics in cells expressing vinculin binding-deficient CAS Y12E was significantly increased (*t*1/2 = 9.1 ± 2.3 s) (Fig. 5a). In contrast to Vin WT, the exchange dynamics of Vin PNSS mutant was not affected by the different CAS tyrosine 12 variants. The half-maximum recovery time after photobleaching GFP-tagged Vin PNSS mutant was 8 ± 3.3 s for CAS WT, 8.8 ± 3.1 s for CAS Y12E, and 9.6 ± 3,7 s for CAS Y12F, which is comparable to the dynamics of Vin WT in CAS Y12E cells (Fig. 5b). These findings are consistent with our previous observations of vinculin dynamics in Src-transformed CAS−/− cells expressing CAS tyrosine 12 variants [27] and suggest that binding to CAS stabilizes vinculin in focal adhesions.

### CAS–vinculin interaction is important for stretch-induced phosphorylation of the CAS substrate domain

**Fig. 6:**
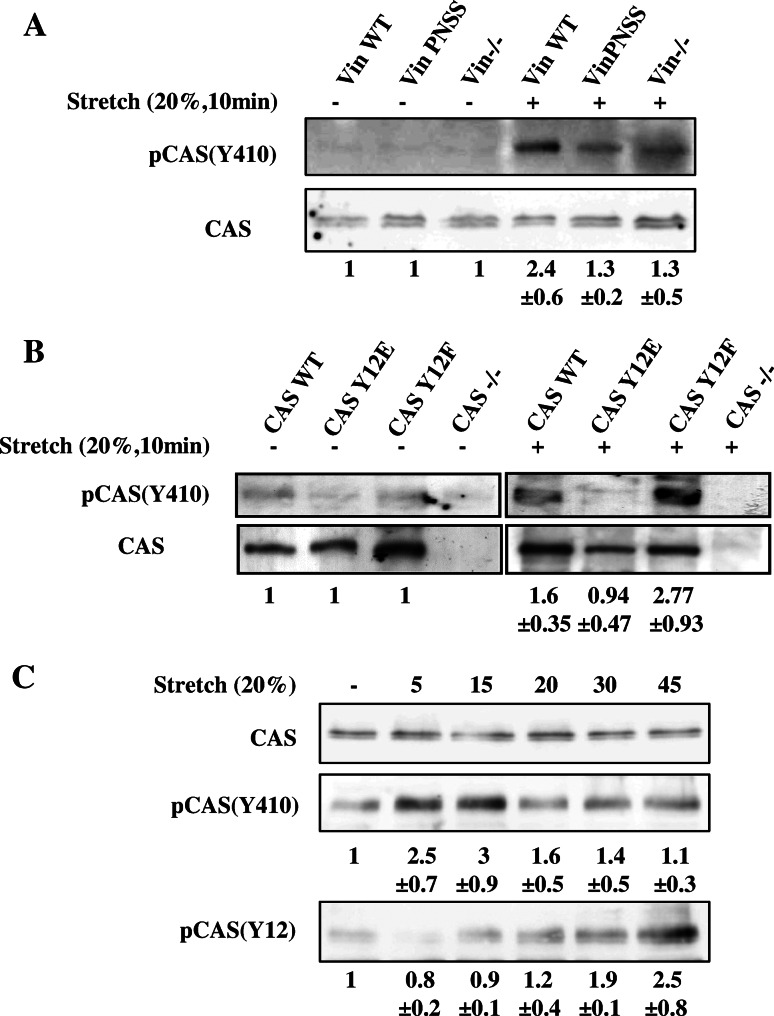
Stretch-mediated phosphorylation of CAS is dependent on CAS–vinculin interaction. **a** Vin−/− MEFs or Vin−/− MEFS re-expressing indicated vinculin variants, **b** CAS−/− MEFs re-expressing indicated CAS variants and **c** MEFs transformed by constitutively active Src (MEFs + Src527F) were seeded on fibronectin-coated flexible membrane, incubated for 24 h, and then subjected to 20 % static stretch for 10 min (**a**, **b**) or for indicated times (**c**). Subsequently, cells were lysed and analyzed by Western blotting against phosphorylated Y410 in the CAS substrate domain and Y12 in CAS SH3 domain. *Numbers* indicate fold-change (mean ± SD) in CAS Y410 and Y12 phosphorylation after stretching. The immunoblots are representative of three independent experiments

The CAS substrate domain has been reported to be extensively phosphorylated in response to mechanical extension of the CAS molecule, both in vivo and in vitro [24]. For the CAS molecule to be extended in response to forces or matrix stretch, it needs to be mechanically anchored on at least two distant sites to cytoskeletal or focal adhesion proteins. We hypothesize that vinculin might serve as one of the anchors. To test whether vinculin binding is necessary for a mechanical activation of CAS, mouse embryonic fibroblasts (MEFs) were cultured on a stretchable silicon substrate and exposed to uniaxial static stretch (20 % for 10 min). Stretch-induced activation of CAS was analyzed by measuring the phosphorylation of the CAS substrate domain at position Y410. We observed only a small stretch-induced increase in phosphorylation of CAS Y410 in cells lacking vinculin or expressing mutated PNSS vinculin that was unable to interact with CAS (Fig. 6a), yet PNSS vinculin retained its ability to reverse high basal phosphorylation of FAK in Vin−/− cells (Fig.S4). The lower FAK phosphorylation at Y397 in PNSS vinculin cells rules out that the stretch-insensitivity of CAS in these cells is an indirect effect mediated by FAK. Moreover, in Vin−/− cells re-expressing WT vinculin, CAS Y410 phosphorylation was significantly increased by 2.4-fold after stretch (Fig. 6a). These data suggest that the direct interaction of vinculin with CAS is indispensable for stretch-induced CAS phosphorylation. Consistently, CAS−/− cells re-expressing CAS carrying Y12E mutation, which blocks CAS–vinculin binding, also prevents stretch-dependent activation of CAS. Furthermore, the CAS Y12F mutation, which enhances vinculin-CAS binding, increases the stretch-dependent activation of CAS (Fig. 6b).

We further analyzed the dynamics of CAS Y410 phosphorylation during stretch. CAS Y410 phosphorylation appears rapidly, peaks after 15 min, and then disappears (Fig. 6c). In contrast, CAS Y12 phosphorylation exhibits different dynamics, characterized by a gradual increase of Y12 phosphorylation during stretch (Fig. 6c).

Taken together, these data indicate that vinculin-CAS interaction is required for stretch-induced CAS substrate domain phosphorylation and supports our hypothesis that vinculin serves as a mechanical coupling protein to transmit forces to the CAS molecule.

### Loss of CAS–vinculin interaction reduces cell stiffness and traction forces, but does not affect adhesion strength

**Fig. 7:**
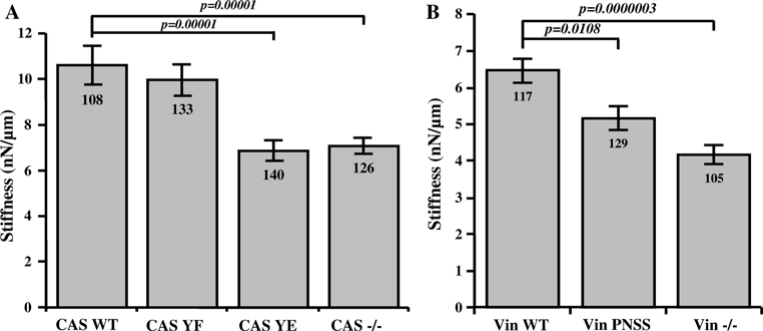
Effect of CAS–vinculin interaction on cell mechanical properties. Stiffness of MEFs analyzed at 6nN force using magnetic tweezers. The *bar* plots show stiffness of **a** CAS−/− MEFs re-expressing indicated CAS variants and **b** Vin−/− MEFs re-expressing indicated vinculin variants.* Numbers in columns* indicate number of analyzed cells, and *error bars* represent standard error

Because vinculin is not only a force-coupling protein but has also been implicated in the regulation of cytoskeletal stiffness and pre-stress [38], we hypothesized that the higher turnover dynamics in cells with impaired CAS–vinculin binding leads to changes in cell stiffness and contractile activation. First, we measured the stiffness of CAS−/− MEF cells re-expressing CAS WT or mutated CAS Y12F and Y12E using magnetic beads coated with fibronectin that were attached to integrin cell surface receptors and pulled laterally with magnetic tweezers. Beads on CAS WT and CAS Y12F cells moved significantly (*p* < 0.05) less in response to lateral forces compared to CAS Y12E. From the bead displacements, we computed the stiffness of cells. CAS WT and CAS Y12F cells are approximately 1.5 times stiffer compared to CAS Y12E cells (Fig. 7a). Next, we measured also the stiffness of Vin−/− MEFs re-expressing Vin WT or mutated Vin PNSS. The stiffness of Vin PNSS cells again was significantly reduced in comparison to Vin WT cells (Fig. 7b).

**Fig. 8:**
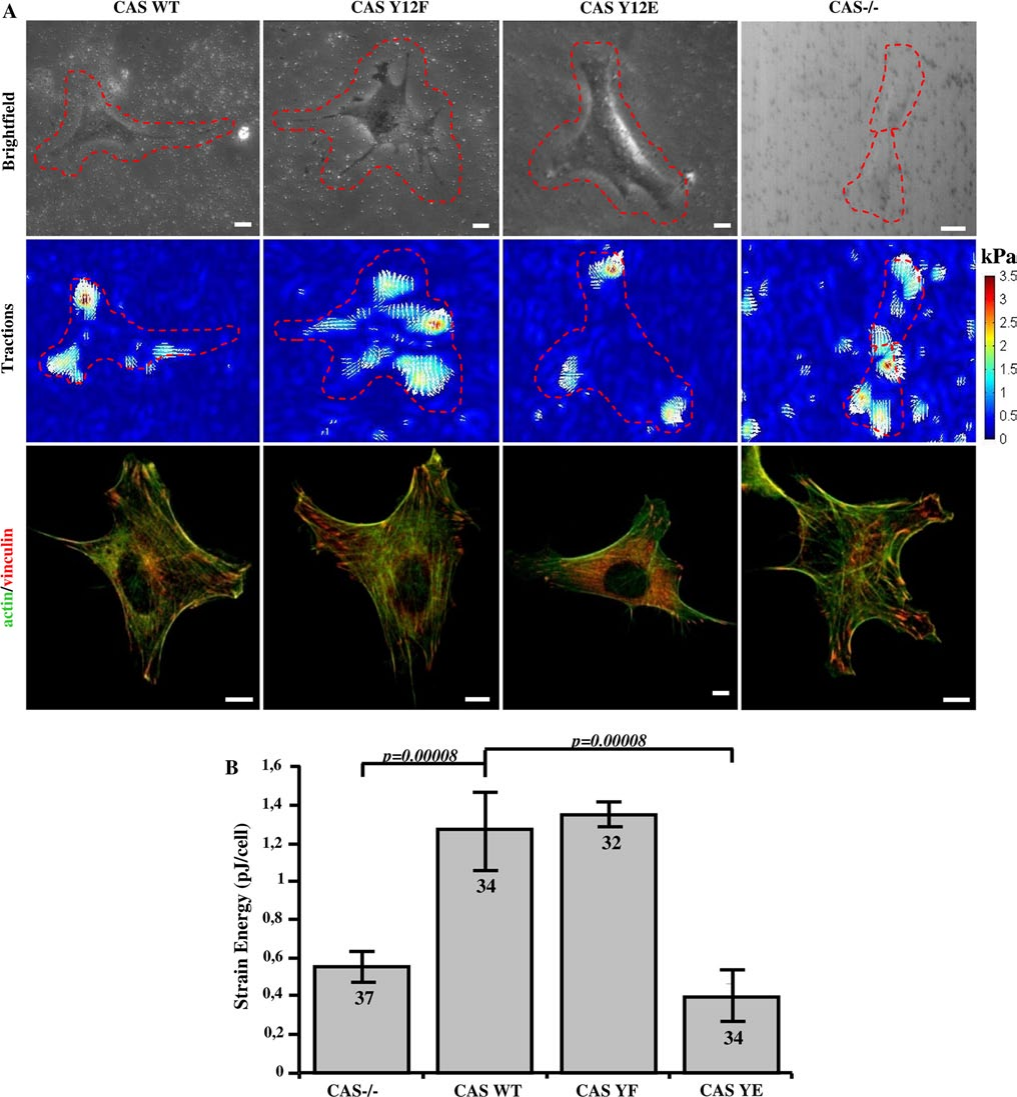
Traction force generation is modulated by phosphorylation of Y12 in the CAS SH3 domain. **a** Bright field (*upper row*), traction field (*middle row*), and fluorescent (*bottom row*) images of CAS−/− MEFs re-expressing indicated CAS variants. *Scale bar* 10 μm. **b** The *bar* *plot* shows the strain energy generated by single cells (mean ± SE).* Numbers in columns* indicate number of analyzed cells

**Fig. 9:**
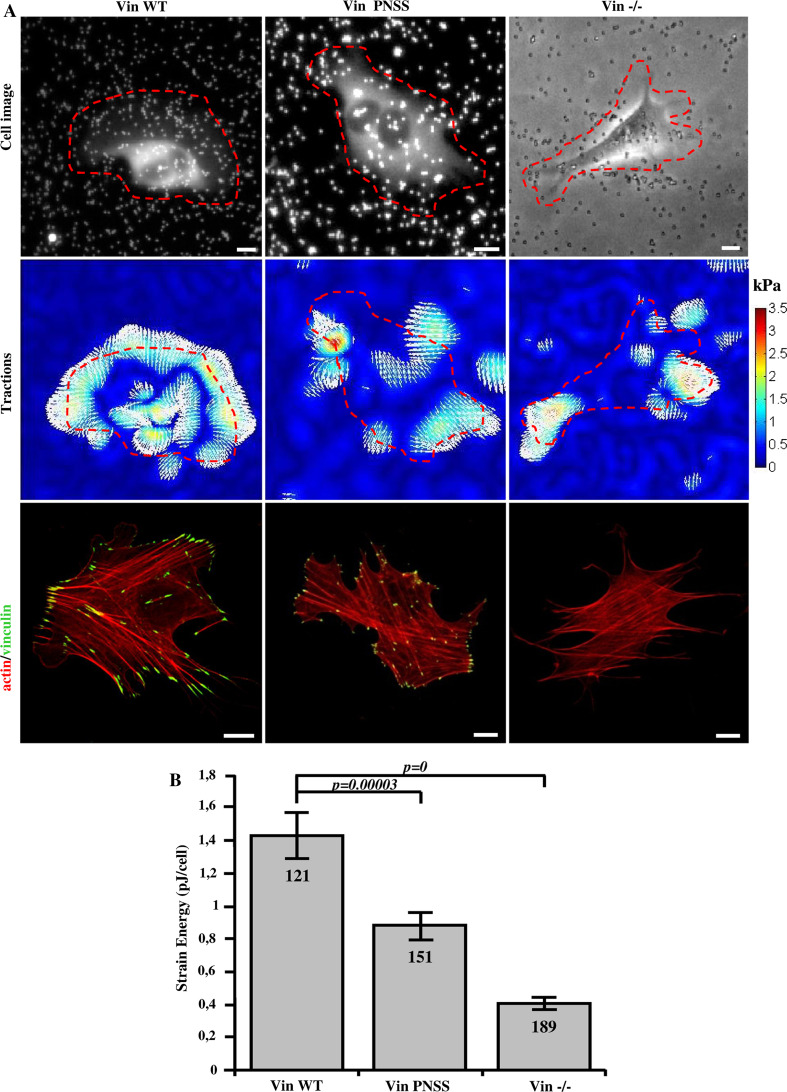
Disruption of CAS–vinculin interaction results in impaired traction forces generation. **a** *Upper row* cell image represented by GFP fluorescence (Vin Wt, Vin PNNS) and bright field image (Vin−/−) traction field (*middle row*), and fluorescent (*bottom row*) images of Vin−/− MEFs re-expressing different vinculin variants. *Scale bar* 10 μm. **b** The *bar* *plot* shows the strain energy generated by single cells (mean ± SE).* Numbers in columns* indicate number of analyzed cells

Because stiffness and contractile pre-stress are linearly related in adherent cells [39], we expected that also the traction forces are reduced in cells with impaired CAS–vinculin binding. The traction field of cells was measured using the Fourier transform traction cytometry method [40] (Fig. 8a). As a scalar value for the traction force magnitude, we computed the elastic strain energy stored in the matrix beneath each cell. The strain energy of vinculin binding-deficient CAS Y12E mutant cells was about three times lower compared to CAS WT and mutant CAS Y12F cells (Fig. 8b). To exclude the possibility that this finding was only due to the impaired FAK-binding to the CAS SH3 domain, we repeated these measurements on CAS binding-deficient Vin PNSS cells and confirmed that traction forces were significantly reduced compared to Vin WT cells (Fig. 9a, b).

To ensure that the lower traction forces of the phospho-mimicking CAS mutants are not caused by diminished adhesion strength, we measured with magnetic tweezers the forces that were needed to detach integrin-bound beads from the cells. The bead binding strength was not markedly different between the CAS WT and CAS Y12 mutant (Y12E, Y12F) cells (Fig. S5). Therefore, the reduced traction forces that we observed in the phospho-mimicking Y12E mutant are not caused by poor adhesion, but are likely due to diminished contractile activation. Taken together, these findings are in support of our hypothesis that proper CAS–vinculin binding is important for vinculin and/or CAS to fulfill its mechano-regulating function.

## Discussion

In this study, we have identified vinculin as a new binding partner of CAS. We have shown that CAS directly interacts with vinculin, and that this interaction is dependent on the CAS SH3 domain and the PKPP sequence of the vinculin’s hinge-region. Vinculin affects the dynamics of CAS within focal adhesions and vice versa. Moreover, vinculin is important for proper CAS localization and targeting in focal adhesions. Disruption of CAS–vinculin interaction leads to decreased cell stiffness and impairs traction force generation. These findings suggest that the interaction of CAS–vinculin is important for regulating the mechanical properties of cells.

### Vinculin PNSS mutation

To analyze CAS–vinculin interaction, we have mutated vinculin in P860**PKPP** to P860**PNSS** and have shown that PNSS mutated vinculin does not bind CAS (Fig. 1e). The P860**PKPP** motif is located within the hinge region of vinculin. Three proline-rich sequences conserved across species can be identified [41] within the hinge region. Looking from the N-terminus, the first F842PPPP motif is distinct from sequences generally recognized by SH3 domains and mediates interaction of vinculin with VASP [42]. The third P871PPRPPPP motif was shown to mediate the interaction of vinculin with Arp2/3 [31] and proteins of the vinexin family [41]. The second AP860**PKPP**LP together with the third motif was suggested to be involved in binding of vinexin family proteins through interaction with a pair of its SH3 domains in tandem [43]. In our study, we identified this second motif as a target binding site of the CAS SH3 domain. The PNSS mutation of the second motif specifically blocks interaction with CAS without significantly affecting the interaction with Arp2/3 or paxillin (Fig. 1e, f). We cannot rule out that mutation of the second motif to PNSS does not affect the binding of proteins of the vinexin family or some unknown vinculin binding partners, however, our results are most consistent with the notion that the inhibition of CAS–vinculin interaction is responsible for the observed phenotypes of the PNSS mutant.

### Vinculin influences targeting of CAS to focal adhesions

Correct assembly and disassembly of focal adhesions is required for regulation of cell migration and proliferation [44]. CAS and vinculin are both important focal adhesion proteins. The interaction between them that we report in this study offers a new insight into the regulation of protein dynamics in focal adhesions. CAS is recruited early to the newly formed adhesions and persists throughout their lifetime [22]. It has been previously shown that the CAS SH3 domain is important for its localization to focal adhesions [22, 27] through coupling to FAK [13, 21]. In this study, we identify vinculin as another important CAS SH3 binding partner. The localization of CAS in focal adhesion, either in FAK−/− or Vin−/− cells, is significantly reduced (Fig. 2), demonstrating that both FAK and vinculin are essential for proper localization of CAS. Since FAK and vinculin localize in different layers of focal adhesions [45], we speculate that CAS may be present throughout the entire focal adhesion complex to integrate signals from different layers. In addition, re-expression of vinculin PNSS, unlike re-expression of vinculin WT in Vin−/− cells, was not able to restore CAS localization to focal adhesions (Fig. 2b, Fig. S3B). This suggests that a direct interaction of CAS with vinculin is necessary for proper localization of CAS to focal adhesions. However, when a form of activated vinculin is re-expressed in Vin−/− cells, CAS localization is restored even when the vinculin lacks the CAS-binding region [46]. This suggests that in the case of focal adhesions stabilized by pre-activated vinculin, either FAK can fully restore the CAS localization to focal adhesions, or the second focal adhesions-targeting domain of CAS, the C-terminal CCH domain [22] can in these conditions gain the ability to compensate for the loss of direct CAS–vinculin interaction.

### Dynamics of CAS and vinculin within focal adhesions

Previously, we have shown that substitution of tyrosine 12 in the CAS SH3 domain to phosphomimicking glutamate results in increased cell migration, and this effect is probably caused by increased focal adhesion dynamics [27]. Vinculin stabilizes focal adhesions and slows down migration [47]. Moreover, the dynamics of vinculin in focal adhesions decreases during the maturation of adhesion contacts [37]. Our FRAP experiments indicate that CAS and vinculin mutually affect their dynamics within focal adhesions, and that this effect is dependent on their direct interaction. CAS dynamics in focal adhesions was decreased when WT vinculin was re-expressed in *Vin*−*/*− cells, whereas re-expression of the mutated (PNSS) vinculin, which can not bind CAS, did not affect CAS dynamics when compared to Vin−/− cells (Fig. 4b). Conversely, the exchange rate of vinculin in focal adhesions was increased in cells expressing CAS Y12E, which cannot interact with vinculin, when compared to cells expressing CAS WT (Fig. 5a). Moreover, the dynamics of the mutated (PNSS) vinculin was not sensitive to expression of Tyr12 CAS variants (CAS WT, Y12E, Y12F) and was similar to WT vinculin dynamics expressed in CAS Y12E cells (Fig. 5b). Taken together, the presence of CAS in the cells and its ability to localize to focal adhesions affects vinculin dynamics. However, the dynamics of the vinculin PNSS mutant (no interaction with CAS) in focal adhesions is neither affected by the presence of CAS nor by the ability of CAS to localize to focal adhesions. These observations are most consistent with the notion that CAS directly affects vinculin dynamics in focal adhesions through a direct interaction with vinculin. In addition, the data suggest that phosphorylation of CAS on Tyr 12 can potentially represent a novel mechanism regulating vinculin dynamics in focal adhesions [27].

Changes in the dynamics of focal adhesion proteins are often associated with changes in the size of adhesion structures. In this study, we have shown that disruption of CAS–vinculin interaction in vinculin-deficient cells and in vinculin PNSS mutant cells leads to a decreased size of adhesive structures (Fig. 3). This is consistent with previous findings that vinculin-deficient cells have smaller focal adhesions [48, 49], and also with our observation of smaller focal adhesions in CAS-deficient cells re-expressing the CAS Y12E mutant (Fig. S6). In contrast, FAK−/− cells have larger focal adhesions when compared to WT MEFs [50] or Vin−/− and CAS−/− cells (see Fig. S3A), suggesting that unlike CAS–vinculin, CAS–FAK interaction is probably not directly involved in the regulation of focal adhesion size.

CAS-mediated effects on vinculin dynamics in focal adhesions and size of focal adhesions may be mechanistically explained by CAS interaction with vinculin that leads to stabilization of vinculin in its open conformation. According to Humphries and colleagues, activated vinculin forms exhibit increased residency time in focal adhesions and increase in size of focal adhesions [51].

### CAS binding to vinculin regulates mechanosensing

Focal adhesions are cellular structures that sense external and internal forces and mechanical properties of the environment. CAS is one of the mechanosensors that is activated by extension and subsequent phosphorylation of tyrosines in the CAS substrate domain [24]. For proper mechanotransduction, CAS has to be anchored on at least two distant sides of the molecule to other force-transmitting proteins. Because vinculin is one of the main force-transmitting proteins in focal adhesions [38, 52, 53], we tested the role of CAS–vinculin interaction for mechanosensing. Stretching experiments revealed an attenuated CAS substrate domain phosphorylation in CAS Y12E transfected cells and also in vinculin PNSS mutant cells, both of which lack CAS–vinculin binding, strongly suggesting that for stretch-dependent phosphorylation of CAS, the interaction of CAS with vinculin is indispensable (Fig. 6).

Previously, we have shown that phosphomimicking mutation of tyrosine 12 (CAS Y12E) delocalizes CAS from focal adhesions [27]. Consistently, we show here that CAS Y12E does not co-localize with vinculin in focal adhesions and that only the CAS WT and CAS Y12F substrate domain was tyrosine phosphorylated in focal adhesions after stretching (Fig. S7). These findings further support our interpretation that a direct interaction of CAS with vinculin is necessary for mechanical stress-mediated CAS substrate domain phosphorylation.

### CAS–vinculin interaction affects cell stiffness and traction force generation

The ability of the cell to resist deformation is important for normal cell function. Cell stiffness is determined by various factors such as number and bond elasticity of force-transmitting and force-generating molecular interactions within the cytoskeleton: between the cytoskeleton and focal adhesions, between cells, and between the cell and the extracellular matrix. We show here that CAS–vinculin interactions participate in these processes through modulating the cytoskeletal prestress. When we applied magnetic pulling forces on CAS Y12E and Vin PNSS cells where CAS–vinculin interaction was inhibited, magnetic beads moved more easily compared to wild-type cells, indicating a lower cell stiffness (Fig. 7). We have previously shown that Src-transformed CAS−/− MEFs expressing CAS Y12E are more invasive in collagen gels than wild-type cells [27], which we attribute to their lower resistance against deformation when navigating through a 3-D environment with a high degree of steric hindrance [54, 55].

In spread cells, cell stiffness is linearly related to cytoskeletal prestress and traction forces [39]. Diminished traction forces can be the result of either reduced actomyosin contractility [56, 57] or reduced adhesion strength [58, 59]. It has been previously shown that loss of vinculin resulted in decreased traction force generation [38, 60], and that the presence of CAS in focal adhesions is crucial for cell contractility [61]. Our data suggest that it is the interaction of both proteins, CAS and vinculin, that is important for traction force generation. CAS–vinculin interactions, however, are not important for maintaining adhesion strength (Fig. S5) even though the size and dynamics of focal adhesions is altered when CAS–vinculin interaction is disrupted. This suggests that the larger traction forces in CAS WT and Y12F cells are facilitated by CAS–vinculin interactions that modulate actomyosin contractility through downstream signaling mechanisms, likely involving the force-dependent activation of CAS.

To summarize, we have identified vinculin as a novel binding partner of CAS, and have shown that CAS–vinculin interactions are important for the internal dynamics of focal adhesion, sensing of mechanical stress, regulating cell stiffness and traction forces.

### CAS-dependent mechanosensing: a possible role of CAS and Src in a negative feedback circuit

CAS was proposed to be a primary mechanical force sensor, transducing forces into mechanical extension of the CAS substrate domain and thereby priming the substrate domain for subsequent activation of downstream signaling [25]. The concept of CAS as a mechanical sensor requires at least two anchoring sites along the CAS structure. The N-terminal SH3 and C-terminal CCH domain, both important for CAS targeting to focal adhesions [22], most likely represent such anchoring domains. Our data suggest that CAS–vinculin interaction is a critical focal adhesion anchoring mechanism for CAS and is essential for CAS-mediated mechanotransduction.

**Fig. 10:**
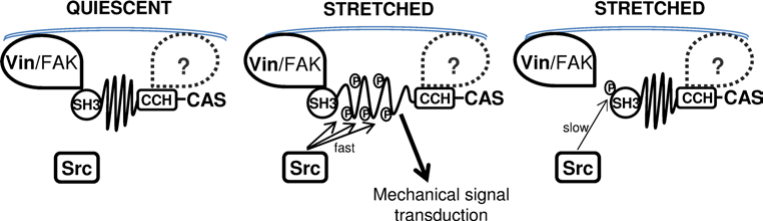
Model for regulation of CAS-dependent mechanosensing. CAS is anchored in focal adhesions by N-terminal SH3 and C-terminal CCH domains [22, 24]. In quiescent cells, the substrate domain of CAS adopts a compact structure (*left*). Mechanical stretch leads to extension of the CAS substrate domain and subsequent phosphorylation by Src, which activates CAS-mediated mechanotransduction signals (*middle*). Src phosphorylation of CAS on tyrosine 12 blocks CAS–vinculin binding, and the substrate domain returns to a compact structure. CAS-mediated mechanotransduction is attenuated (*right*) either by a gradual loss of substrate domain phosphorylation or by making the phosphorylated tyrosines in the substrate domain inaccessible for downstream signaling proteins

CAS-mediated mechanotransduction in turn requires phosphorylation of the CAS substrate domain by Src [25]. At the same time, Src also phosphorylates CAS on tyrosine 12 and inhibits CAS binding not only to FAK and PTP–PEST [27] but also to vinculin, as demonstrated by the phosphomimicking Y12E mutation (Fig. 1b). Results from a structural modeling study confirm that phosphorylation of tyrosines responsible for substrate binding of SH3 domains (in CAS Y12) in general reduces the binding to SH3 domain interacting partners [28]. The implication is that CAS phosphorylation on tyrosine 12 by Src can inhibit the binding to vinculin and hence prevent the mechanical extension of the CAS substrate domain. This raises the possibility that Src both initiates and terminates CAS-mediated mechanotransduction in a negative feedback loop (Fig. 10).

The CAS substrate domain tyrosine phosphorylation and phosphorylation of tyrosine 12 exhibit significantly different dynamics (Fig. 6c). While the substrate domain phosphorylation is rapid and returns to basal levels 30 min after the onset of stretch, phosphorylation on tyrosine 12 is gradual and becomes noticeably elevated only 20 min after the onset of stretch. The faster dynamics of tyrosine phosphorylation in the mechanically extended substrate domain versus phosphorylation of tyrosine 12 could reflect that the latter is a much weaker phosphorylation motif for Src kinase and also less accessible. The observation of different dynamics of substrate domain and tyrosine 12 phosphorylation supports the notion of a dual role of Src in CAS-mediated mechanotransduction. Mechanical stretch of the cell is transmitted through focal adhesions to the CAS substrate domain, which becomes physically extended, undergoes phosphorylation by Src, and subsequently leads to the activation of CAS-mediated mechanotransduction pathways. Later, the loss of CAS–vinculin association after tyrosine 12 eventually becomes phosphorylated (Fig. 6c) releases the SH3-mediated anchoring of CAS to focal adhesions and reverse the CAS substrate domain to its unextended state. This in turn can be expected to inhibit CAS-mediated signaling either by gradual loss of substrate domain phosphorylation, or by making the substrate domain inaccessible for downstream signaling proteins. Hence, a Src-mediated negative feedback loop would imply that CAS signaling during a static mechanical stretch is only transient.

## Materials and methods

### Cell transfection and culture

CAS−*/*− and FAK−/− MEFs were obtained from Steven Hanks (Vanderbilt University, Nashville). Wild-type MEFs and Vin−/− cells were a kind gift from Dr. W.H. Ziegler, University of Leipzig. CAS−/− MEFs stably re-expressing CAS Tyr12 variants (WT, Y12E, Y12F), were prepared using pIRES3-puro-CAS vector variants as described previously [27]. All transfections were carried out according to the manufacturer’s protocol using Jet Prime (Polyplus Transfection). CAS−/− were transfected with pEGFP-C1 CAS [27] or pcDNA3.1- EGFP-vinculin [62]. Vin−/− MEFs were transfected with pcDNA3.1- EGFP-vinculin and pCS2-wtCAS-venus [22]. FAK−/− cells were transfected with pCS2-wtCAS-venus. All cell lines were cultivated in full DMEM (Life Technologies) with 4.5 g/l L-glucose, L-glutamine, and pyruvate, supplemented with 10 % fetal bovine serum (Sigma-Aldrich, St. Louis, MO, USA), 2 % antibiotic–antimycotic (Life Technologies), and 1 % MEM non-essential amino acids (Life Technologies). To abrogate the **PKP**P CAS SH3-binding motif in vinculin, we mutagenized the vinculin cDNA using QuikChange II Kit (Stratagene). The resulting construct was verified by sequencing.

### Immunoblotting and immunoprecipitation

Subconfluent cell cultures were washed with phosphate-buffered saline (PBS) and lysed in modified RIPA buffer (0.15 M NaCl; 50 mM Tris-HCl, pH 7.4; 1 % Nonidet P-40; 0.1 % SDS; 1 % sodium deoxycholate; 5 mM EDTA; 50 mM NaF). Protein concentrations in lysates were determined using the DC Protein Assay (Bio-Rad, Hercules, CA, USA). Protein lysates were diluted in Laemmli sample buffer (0.35 M Tris-HCl, pH 6.8; 10 % SDS; 40 % glycerol; 0.012 % Bromophenol blue). For immunoblotting, samples were separated on 10 % SDS-polyacrylamide gels and transferred onto nitrocellulose membranes. Non-specific activity was blocked by incubating membranes for 45 min at room temperature in Tris-buffered saline containing 5 % non-fat dry milk. Membranes were then incubated overnight at 4 °C with primary antibodies, washed extensively with Tris-buffered saline with Tween-20 (TTBS), and incubated for 1 h at room temperature with horseradish peroxidase (HRP)—conjugated secondary antibodies. After extensive washing in TTBS, the blots were developed using the LAS-1000 Single System (Fujifilm, Tokyo, Japan). The monoclonal antibody against CAS (clone 24) was obtained from BD Transduction Laboratories. Anti-vinculin monoclonal antibodies were from Sigma Aldrich. Anti-FAK polyclonal rabbit antibodies (FAK C-20), anti-GFP (sc8334), and HRP-conjugated anti-mouse and anti–rabbit immunoglobulin G were purchased from Santa Cruz Biotechnology. Phospho-specific antibodies against CAS phosphotyrosine 410 were bought from Cell Signaling Technology. Antibody against CAS phosphotyrosine 12 was developed recently [27]. Quantification of Western blots was carried out using ImageJ software (http://rsbweb.nih.gov/ij/).

For immunoprecipitations, cells were lysed in NP-40 lysis buffer (50 mM Tris, pH 7.4, 150 mM NaCl, 1 % NP-40, 5 mM EDTA, 50 mM NaF). Lysates containing 500 mg of proteins were incubated overnight on ice with 1 μg of primary antibody (monoclonal anti-GFP antibody; Molecular Probes, Invitrogen), and immune complexes were collected by additional 2-h incubation with protein A–Sepharose (20 μl of 50 % slurry; Zymed, San Francisco, CA, USA). The immunoprecipitates were washed five times with 1 ml of ice-cold NP-40 lysis buffer, resuspended in 2× SDS-PAGE sample buffer, and processed for immunoblotting.

### Far-Western-blot analysis

Cell lysates were prepared from Vinculin−/− MEFs re-expressing Vinculin WT tagged with GFP in a NP-40 lysis buffer (50 mM Tris, pH 7.4, 150 mM NaCl, 1 % NP-40, 5 mM EDTA, 50 mM NaF). Protein samples were prepared as described under “Immunoprecipitations”. After SDS-PAGE, proteins were transferred to nitrocellulose membrane and far-Western-blot analysis was carried out by incubating the protein blots with the recombinant protein probe at 1.2 μg/ml and anti-GST antibody (Sigma, 1:4000) in 1 % BSA in TTBS overnight. After extensive washing with TTBS, blots were treated with horseradish peroxidase-conjugated secondary antibodies and the blots were developed using the LAS-1000 Single System (Fujifilm, Tokyo, Japan).

### GST pull-downs and MS analysis

CAS SH3 domain and mutational variants with either the non-phosphorylatable Y12F substitution or a negative control phospho-mimicking Y12E substitution were N-terminally fused with glutathione-S-transferase. The recombinant protein was purified and incubated with HeLa cell lysate by applying an affinity chromatography strategy. The interacting proteins were separated by SDS-PAGE, and proteins differentially bound by both WT and Y12F CAS–SH3 domain variants were identified by peptide mass fingerprinting using a 4800 Plus MALDI TOF/TOF (AB SCIEX) mass spectrometer.

Cell lysates were incubated with glutathione Sepharose 4B beads with immobilized GST or GST–CAS–SH3 variants at 4 °C for 2 h. The beads were washed extensively and boiled in Laemmli sample buffer, and proteins were detected by SDS-PAGE and immunoblotting.

### Immunofluorescence microscopy

Cells were seeded either on coverslips or on PDMS membranes (for stretching), both coated with human 5 μg/ml fibronectin (Invitrogen, Carlsbad, CA, USA). Cells seeded on coverslips were grown for 24–48 h, and subsequently fixed in 4 % paraformaldehyde, permeabilized in 0.5 % Triton X-100, washed extensively with PBS, and blocked in 3 % bovine serum albumin. Cells attached to PDMS membranes were stretched for 10 min at 0.25 Hz and 20 % peak-to-peak amplitude, immediately fixed in 4 % paraformaldehyde, permeabilized in 0.5 % Triton X-100, washed with PBS, and blocked in 3 % bovine serum albumin. PDMS membranes with cells were then mounted on coverslips. The cells (both on coverslips and PDMS membranes) were sequentially incubated with primary antibodies for 2 h, secondary antibodies for 60 min, and if needed with Dy-405 phalloidin (Dyomics) for 15 min, and extensive washing between each step. The primary antibodies were as follows: anti-vinculin (Jackson Biotechnology), anti-paxillin (BD Transduction Laboratories). The secondary antibodies were as follows: anti-rabbit (Alexa 546) and anti-mouse (Alexa 594, Alexa 633; Molecular Probes). Images were acquired with a TCS SP2 microscope system (Leica, Wetzlar, Germany) equipped with a Leica 63×/1.45 oil objective.

### Fluorescence recovery after photobleaching

FRAP studies were conducted on live cells expressing either venus-tagged CAS or GFP-tagged vinculin. The cells were placed on glass-bottom dishes (MatTek, Ashland, MA, USA) coated with 10 μg/ml fibronectin and cultured for 24 h before the experiment. Measurements were performed in DMEM at 37 °C and 5 % CO2 and 12–18 focal adhesions from different cells, expressing CAS-venus or GFP-vinculin were analyzed. After a brief measurement for monitoring baseline intensity (488 nm), a high-energy beam was used to bleach 40–80 % of the intensity in the spot. The intensity of recovery of the bleached region was extracted from the images series, and curves were fitted to a single-exponential using SigmaPlot (SYSTAT Software). The characteristic fluorescence recovery time was extracted from the FRAP curves as described by Tolde et al. [63].

### Cell stretcher

Stretch experiments were carried out either on flexible polydimethylsiloxane (PDMS, Sylgard) substrates that were molded into the shape of a cell culture well or on commercial silicone chambers (*B* Bridge International) all with 4.0 cm^2^ internal surface [64]. The stretchers for both types of chambers consist of a linear stage for uniaxial stretch and are driven by a computer controlled stepper motor. The substrates were then coated with 5 μg/ml fibronectin in PBS overnight at 4 °C, and 10,000 cells were seeded 24 h prior to experiments. Uniaxial stretch was performed in the incubator under normal cell culture conditions (37 °C, 5 % CO_2_, 95 % humidity) for 10 min at 20 % stretch amplitude (peak-to-peak) [65].

### Traction microscopy

Traction measurements were performed on 7.0 % acrylamide/bisacrylamide (ratio 29:1) gels (Young’s modulus 18.0 kPa, thickness 300 μm) with 1.0 μm red fluorescent beads embedded at the top surface [66, 67]. Gels were coated with 5 μg/ml fibronectin at 4 °C overnight. Cells were seeded on the gels at a density of 5,000 cells per cm^2^ and incubated under normal growth conditions overnight. During measurements, the cells were maintained at 37 °C and 5 % CO_2_ in a humidified atmosphere. Cell tractions were computed by an unconstrained fast Fourier traction cytometry method [40] and measured before and after the cells were treated with 80 μM cytochalasin D to relax the traction forces.

### Magnetic tweezers experiments

We used high-force magnetic tweezers as described in [68]. For measurements, 30,000 cells were seeded overnight in a 35-mm tissue culture dish. Thirty minutes prior to experiments, cells were incubated with 4.5-μm fibronectin-coated superparamagnetic beads (Invitrogen, Karlsruhe, Germany). The tip of the magnetic tweezers was then placed at a distance of 20–30 μm from a bead bound to a cell. Transfected cells were identified in fluorescence mode. During measurements, bright field images were taken at 40× magnification (NA 0.6) with a CCD camera (ORCA ER, Hamamatsu) at a rate of 40 frames/s. The bead position was tracked using an intensity-weighted center-of-mass algorithm. Measurements on multiple beads per well were performed at 37 °C for a maximum duration of 30 min.

### Statistical analysis

Statistical analysis was performed with an unpaired two-tailed Student’s *t* test. Numbers indicate actual *p* value for particular test.

### Electronic supplementary material

Below is the link to the electronic supplementary material.

GST fused WT SH3 domain was used to pull down vinculin from either whole cell or FAK depleted lysates. After immunoblotting, FAK and vinculin were detected by anti-FAK and anti-vinculin antibodies. (TIFF 170 kb)

CAS and vinculin co-localize in focal adhesions. MEFs expressing GFP CAS WT, Y12E and Y12F (green) were grown for 24 h on fibronectin-coated coverslips and subsequently stained for vinculin (red). CAS and vinculin localization was determined by confocal fluorescence microscopy. Scale bar: 10 μm. (TIFF 3826 kb)

Dependence of CAS localization in focal adhesions. (A) CAS−/−, FAK−/−, Vin−/− MEFs re-expressing GFP CAS WT were grown on fibronectin-coated coverslips and stained for paxillin (focal adhesion marker) and F-actin. CAS localization was determined by confocal fluorescence microscopy. Scale bar: 10 μm. (B) Vin−/− MEFs expressing mCherry CAS WT alone or with either GFP Vin WT or GFP Vin PNSS were grown on fibronectin-coated coverslips and stained for F-actin (and paxillin in case of Vin−/−). CAS localization was determined by confocal fluorescence microscopy. Scale bar: 10 μm (TIFF 3535 kb)

Effect of vinculin on FAK phosphorylation. Vin−/− MEFs or Vin−/− MEFs re-expressing indicated vinculin variants were analyzed using a phosphospecific antibody (anti-FAK Y397) and total anti-FAK antibody. Amount of vinculin was determined by total vinculin antibody. Numbers indicate average ratios between phosphorylated and total FAK normalized to Vin WT from three independent experiments. (TIFF 139 kb)

Effect of CAS–vinculin interaction on adhesion strength. The curves show the dependence of fibronectin-coated bead detachment (expressed as cumulative rupture probability) on pulling force in different CAS mutant cells. (TIFF 156 kb)

Effect of tyrosine 12 phosphorylation in the CAS SH3 domain on focal adhesion size. CAS−/− MEFs re-expressing CAS Y12 variants (WT, Y12E, Y12F) were grown on fibronectin-coated coverslips and stained for paxillin (focal adhesion marker). The focal adhesion size was determined using confocal microscopy. The bar plot shows average size of adhesion structures in CAS−/− cells or cells re-expressing indicated CAS variant. Numbers in columns indicate number of analyzed focal adhesions. Error bars represent standard errors. (TIFF 240 kb)

Phosphorylated CAS co-localizes with vinculin in focal adhesions after stretch. CAS−/− MEFs re-expressing indicated CAS variants were seeded on fibronectin-coated PDMS stretchable membranes, incubated for 24 h, and subjected to 20 % static stretch for 10 min. Co-localization of CAS and vinculin was analyzed by confocal fluorescence microscopy. Arrows are pointing at the adhesion sites where phosphorylated CAS co-localizes with vinculin. Scale bar: 10 μm. (TIFF 2308 kb)

Supplementary material 8 (TIFF 204 kb)

## References

[1] Defilippi P, Di Stefano P, Cabodi S (2006). p130Cas: a versatile scaffold in signaling networks. Trends Cell Biol.

[2] Brabek J, Constancio BS, Shin NY, Pozzi A, Weaver AM, Hanks SK (2004). CAS promotes invasiveness of Src-transformed cells. Oncogene.

[3] Brabek J, Constancio SS, Siesser PF, Shin NY, Pozzi A, Hanks SK (2005). Crk-associated substrate tyrosine phosphorylation sites are critical for invasion and metastasis of Src-transformed cells. Mol Cancer Res.

[4] Brinkman A, van der Flier S, Kok EM, Dorssers LCJ (2000). BCAR1, a human homologue of the adapter protein p130Cas, and antiestrogen resistance in breast cancer cells. J Natl Cancer Inst.

[5] Dorssers LCJ, Grebenchtchikov N, Brinkman A, Look MP, Klijn AGM, Geurts-Moespot A, Span PN, Foekens JA, Sweep CGJ (2004). Application of a newly developed ELISA for BCAR1 protein for prediction of clinical benefit of tamoxifen therapy in patients with advanced breast cancer. Clin Chem.

[6] Ruest PJ, Shin NY, Polte TR, Zhang X, Hanks SK (2001). Mechanisms of CAS substrate domain tyrosine phosphorylation by FAK and Src. Mol Cell Biol.

[7] Shin NY, Dise RS, Schneider-Mergener J, Ritchie MD, Kilkenny DM, Hanks SK (2004). Subsets of the major tyrosine phosphorylation sites in Crk-associated substrate (CAS) are sufficient to promote cell migration. J Biol Chem.

[8] Fonseca PM, Shin NY, Brabek J, Ryzhova L, Wu J, Hanks SK (2004). Regulation and localization of CAS substrate domain tyrosine phosphorylation. Cell Signal.

[9] Klemke RL, Leng J, Molander R, Brooks PC, Vuori K, Cheresh DA (1998). CAS/Crk coupling serves as a “molecular switch” for induction of cell migration. J Cell Biol.

[10] Honda H, Nakamoto T, Sakai R, Hirai H (1999). p130(Cas), an assembling molecule of actin filaments, promotes cell movement, cell migration, and cell spreading in fibroblasts. Biochem Biophys Res Commun.

[11] Cho SY, Klemke RL (2000). Extracellular-regulated kinase activation and CAS/Crk coupling regulate cell migration and suppress apoptosis during invasion of the extracellular matrix. J Cell Biol.

[12] Huang JH, Hamasaki H, Nakamoto T, Honda H, Hirai H, Saito M, Takato T, Sakai R (2002). Differential regulation of cell migration, actin stress fiber organization, and cell transformation by functional domains of Crk-associated substrate. J Biol Chem.

[13] Polte TR, Hanks SK (1995). Interaction between focal adhesion kinase and Crk-associated tyrosine kinase substrate P130(Cas). Proc Natl Acad Sci USA.

[14] Li X, Earp HS (1997). Paxillin is tyrosine-phosphorylated by and preferentially associates with the calcium-dependent tyrosine kinase in rat liver epithelial cells. J Biol Chem.

[15] Harte MT, Hildebrand JD, Burnham MR, Bouton AH, Parsons JT (1996). p130(Cas), a substrate associated with v-Src and v-Crk, localizes to focal adhesions and binds to focal adhesion kinase. J Biol Chem.

[16] Garton AJ, Burnham MR, Bouton AH, Tonks NK (1997). Association of PTP-PEST with the SH3 domain of p130(cas); a novel mechanism of protein tyrosine phosphatase substrate recognition. Oncogene.

[17] Kirsch KH, Georgescu MM, Hanafusa H (1998). Direct binding of p130(Cas) to the guanine nucleotide exchange factor C3G. J Biol Chem.

[18] Liu F, Sells MA, Chernoff J (1998). Transformation suppression by protein tyrosine phosphatase 1B requires a functional SH3 ligand. Mol Cell Biol.

[19] Kirsch KH, Georgescu MM, Ishimaru S, Hanfusa H (1999). CMS: an adapter molecule involved in cytoskeletal rearrangements. Proc Natl Acad Sci USA.

[20] Nakamoto T, Yamagata T, Sakai R, Ogawa S, Honda H, Ueno H, Hirano N, Yazaki Y, Hirai H (2000). CIZ, a zinc finger protein that interacts with p130(cas) and activates the expression of matrix metalloproteinases. Mol Cell Biol.

[21] Polte TR, Hanks SK (1997). Complexes of focal adhesion kinase (FAK) and Crk-associated substrate (p130(Cas)) are elevated in cytoskeleton-associated fractions following adhesion and Src transformation—requirements for Src kinase activity and FAK proline-rich motifs. J Biol Chem.

[22] Donato DM, Ryzhova LM, Meenderink LM, Kaverina I, Hanks SK (2010). Dynamics and mechanism of p130Cas localization to focal adhesions. J Biol Chem.

[23] Nakamoto T, Sakai R, Honda H, Ogawa S, Ueno H, Suzuki T, Aizawa S, Yazaki Y, Hirai H (1997). Requirements for localization of p130(cas) to focal adhesions. Mol Cell Biol.

[24] Sawada Y, Tamada M, Dubin-Thaler BJ, Cherniavskaya O, Sakai R, Tanaka S, Sheetz MP (2006). Force sensing by mechanical extension of the Src family kinase substrate p130Cas. Cell.

[25] Sawada Y, Nakamura K, Doi K, Takeda K, Tobiume K, Saitoh M, Morita K, Komuro I, De Vos K, Sheetz M, Ichijo H (2001). Rap1 is involved in cell stretching modulation of p38 but not ERK or JNK MAP kinase. J Cell Sci.

[26] Tamada M, Sheetz MP, Sawada Y (2004). Activation of a signaling cascade by cytoskeleton stretch. Dev Cell.

[27] Janostiak R, Tolde O, Bruhova Z, Novotny M, Hanks SK, Rosel D, Brabek J (2011). Tyrosine phosphorylation within the SH3 domain regulates CAS subcellular localization, cell migration, and invasiveness. Mol Biol Cell.

[28] Tatarova Z, Brabek J, Rosel D, Novotny M (2012) SH3 domain tyrosine phosphorylation—sites, role and evolution. PLoS One 7: Article ID e36310 10.1371/journal.pone.0036310 PMC3352900 22615764

[29] Coutu MD, Craig SW (1988). cDNA-derived sequence of chicken-embryo vinculin. Proc Natl Acad Sci USA.

[30] Bubeck P, Pistor S, Wehland J, Jockusch BM (1997). Ligand recruitment by vinculin domains in transfected cells. J Cell Sci.

[31] Demali KA, Barlow CA, Burridge K (2002). Recruitment of the Arp2/3 complex to vinculin: coupling membrane protrusion to matrix adhesion. J Cell Biol.

[32] Geiger B, Bershadsky A, Pankov R, Yamada KM (2001). Transmembrane extracellular matrix-cytoskeleton crosstalk. Nat Rev Mol Cell Biol.

[33] Subauste MC, Pertz O, Adamson ED, Turner CE, Junger S, Hahn KM (2004). Vinculin modulation of paxillin-FAK interactions regulates ERK to control survival and motility. J Cell Biol.

[34] Giannone G, Ronde P, Gaire M, Beaudouin J, Haiech J, Ellenberg J, Takeda K (2004). Calcium rises locally trigger focal adhesion disassembly and enhance residency of focal adhesion kinase at focal adhesions. J Biol Chem.

[35] Cluzel C, Saltel F, Lussi J, Paulhe F, Imhof BA, Wehrle-Haller B (2005). The mechanisms and dynamics of alpha v beta 3 integrin clustering in living cells. J Cell Biol.

[36] Goetz JG (2009). Bidirectional control of the inner dynamics of focal adhesions promotes cell migration. Cell Adh Migr.

[37] Möhl CC, Kirchgessner N, Schafer C, Kupper K, Born S, Diez G, Goldmann WH, Merkel R, Hoffmann B (2009). Becoming stable and strong: the interplay between vinculin exchange dynamics and adhesion strength during adhesion site maturation. Cell Motil Cytoskelet.

[38] Mierke CT, Kollmannsberger P, Zitterbart DP, Smith J, Fabry B, Goldmann WH (2008). Mechano-coupling and regulation of contractility by the vinculin tail domain. Biophys J.

[39] Wang N, Tolic-Norrelykke IM, Chen JX, Mijailovich SM, Butler JP, Fredberg JJ, Stamenovic D (2002). Cell prestress. I. Stiffness and prestress are closely associated in adherent contractile cells. Am J Physiol Cell Physiol.

[40] Butler JP, Tolic-Norrelykke IM, Fabry B, Fredberg JJ (2002). Traction fields, moments, and strain energy that cells exert on their surroundings. Am J Physiol Cell Physiol.

[41] Zhang JH, Li X, Yao B, Shen WQ, Sun HB, Xu C, Wu JH, Shi YY (2007). Solution structure of the first SH3 domain of human vinexin and its interaction with vinculin peptides. Biochem Biophys Res Commun.

[42] Huttelmaier S, Mayboroda O, Harbeck B, Jarchau T, Jockusch BM, Rudiger M (1998). The interaction of the cell-contact proteins VASP and vinculin is regulated by phosphatidylinositol-4,5-bisphosphate. Current Biol.

[43] Kioka N, Sakata S, Kawauchi T, Amachi T, Akiyama SK, Okazaki K, Yaen C, Yamada KM, Aota S (1999). Vinexin: a novel vinculin-binding protein with multiple SH3 domains enhances actin cytoskeletal organization. J Cell Biol.

[44] Webb DJ, Donais K, Whitmore LA, Thomas SM, Turner CE, Parsons JT, Horwitz AF (2004). FAK-Src signalling through paxillin, ERK and MLCK regulates adhesion disassembly. Nat Cell Biol.

[45] Kanchanawong P, Shtengel G, Pasapera AM, Ramko EB, Davidson MW, Hess HF, Waterman CM (2010). Nanoscale architecture of integrin-based cell adhesions. Nature.

[46] Carisey A, Tsang R, Greiner AM, Nijenhuis N, Heath N, Nazgiewicz A, Kemkemer R, Derby B, Spatz J, Ballestrem C (2013). Vinculin regulates the recruitment and release of core focal adhesion proteins in a force-dependent manner. Curr Biol.

[47] Saunders RM, Holt MR, Jennings L, Sutton DH, Barsukov IL, Bobkov A, Liddington RC, Adamson EA, Dunn GA, Critchley DR (2006). Role of vinculin in regulating focal adhesion turnover. Eur J Cell Biol.

[48] Fernandez JLR, Geiger B, Salomon D, BenZeev A (1993). Suppression of vinculin expression by antisense transfection confers changes in cell morphology, motility, and anchorage-dependent growth of 3T3-cells. J Cell Biol.

[49] Goldmann WH, Schindl M, Cardozo TJ, Ezzell RM (1995). Motility of vinculin-deficient F9 embryonic carcinoma-cells analyzed by video, laser confocal, and reflection interference contrast microscopy. Exp Cell Res.

[50] Ilic D, Furuta Y, Kanazawa S, Takeda N, Sobue K, Nakatsuji N, Nomura S, Fujimoto J, Okada M, Yamamoto T (1995). Reduced cell motility and enhanced focal adhesion contact formation in cells from FAK-deficient mice. Nature.

[51] Humphries JD, Wang P, Streuli C, Geiger B, Humphries MJ, Ballestrem C (2007). Vinculin controls focal adhesion formation by direct interactions with talin and actin. J Cell Biol.

[52] Diez G, Kollmannsberger P, Mierke CT, Koch TM, Vali H, Fabry B, Goldmann WH (2009). Anchorage of vinculin to lipid membranes influences cell mechanical properties. Biophys J.

[53] Ezzell RM, Goldmann WH, Wang N, Parashurama N, Ingber DE (1997). Vinculin promotes cell spreading by mechanically coupling integrins to the cytoskeleton. Exp Cell Res.

[54] Cross SE, Jin YS, Rao J, Gimzewski JK (2007). Nanomechanical analysis of cells from cancer patients. Nat Nanotechnol.

[55] Swaminathan V, Mythreye K, O’Brien ET, Berchuck A, Blobe GC, Superfine R (2011). Mechanical stiffness grades metastatic potential in patient tumor cells and in cancer cell lines. Cancer Res.

[56] Wakatsuki T, Schwab B, Thompson NC, Elson EL (2001). Effects of cytochalasin D and latrunculin B on mechanical properties of cells. J Cell Sci.

[57] Beningo KA, Hamao K, Dembo M, Wang YL, Hosoya H (2006). Traction forces of fibroblasts are regulated by the Rho-dependent kinase but not by the myosin light chain kinase. Arch Biochem Biophys.

[58] Zhang X, Jiang G, Cai Y, Monkley SJ, Critchley DR, Sheetz MP (2008). Talin depletion reveals independence of initial cell spreading from integrin activation and traction. Nat Cell Biol.

[59] Dey T, Mann MC, Goldmann WH (2011). Comparing mechano-transduction in fibroblasts deficient of focal adhesion proteins. Biochem Biophys Res Commun.

[60] Mierke CT, Kollmannsberger P, Zitterbart DP, Diez G, Koch TM, Marg S, Ziegler WH, Goldmann WH, Fabry B (2010). Vinculin facilitates cell invasion into three-dimensional collagen matrices. J Biol Chem.

[61] Tang DD, Tan J (2003). Role of Crk-associated substrate in the regulation of vascular smooth muscle contraction. Hypertension.

[62] Diez G, Auernheimer V, Fabry B, Goldmann WH (2011). Head/tail interaction of vinculin influences cell mechanical behavior. Biochem Biophys Res Commun.

[63] Tolde O, Rosel D, Janostiak R, Vesely P, Brabek J (2012). Dynamics and morphology of focal adhesions in complex 3D environment. Folia Biol.

[64] Faust U, Hampe N, Rubner W, Kirchgessner N, Safran S, Hoffmann B, Merkel R (2011). Cyclic stress at mHz frequencies aligns fibroblasts in direction of zero strain. PLoS One.

[65] Bonakdar N, Luczak J, Lautscham L, Czonstke M, Koch TM, Mainka A, Jungbauer T, Goldmann WH, Schroder R, Fabry B (2012). Biomechanical characterization of a desminopathy in primary human myoblasts. Biochem Biophys Res Commun.

[66] Pelham RJ, Wang YL (1998). Cell locomotion and focal adhesions are regulated by substrate flexibility (vol 94, pg 13661, 1997). Proc Natl Acad Sci USA.

[67] Raupach C, Zitterbart DP, Mierke CT, Metzner C, Muller FA., Fabry B (2007) Stress fluctuations and motion of cytoskeletal-bound markers. Phys Rev 76: Article ID 011918 10.1103/PhysRevE.76.011918 17677505

[68] Kollmannsberger P, Fabry B (2007) High-force magnetic tweezers with force feedback for biological applications. Rev Sci Instrum 78: Article ID 114301 10.1063/1.2804771 18052492

