# Supplementary figures and images for: CAS directly interacts with vinculin to control mechanosensing and focal adhesion dynamics
Source: Cell Mol Life Sci. 2013 Aug 25;71(4):727–44. doi: 10.1007/s00018-013-1450-x (PMC3901934; doi:10.1007/s00018-013-1450-x)

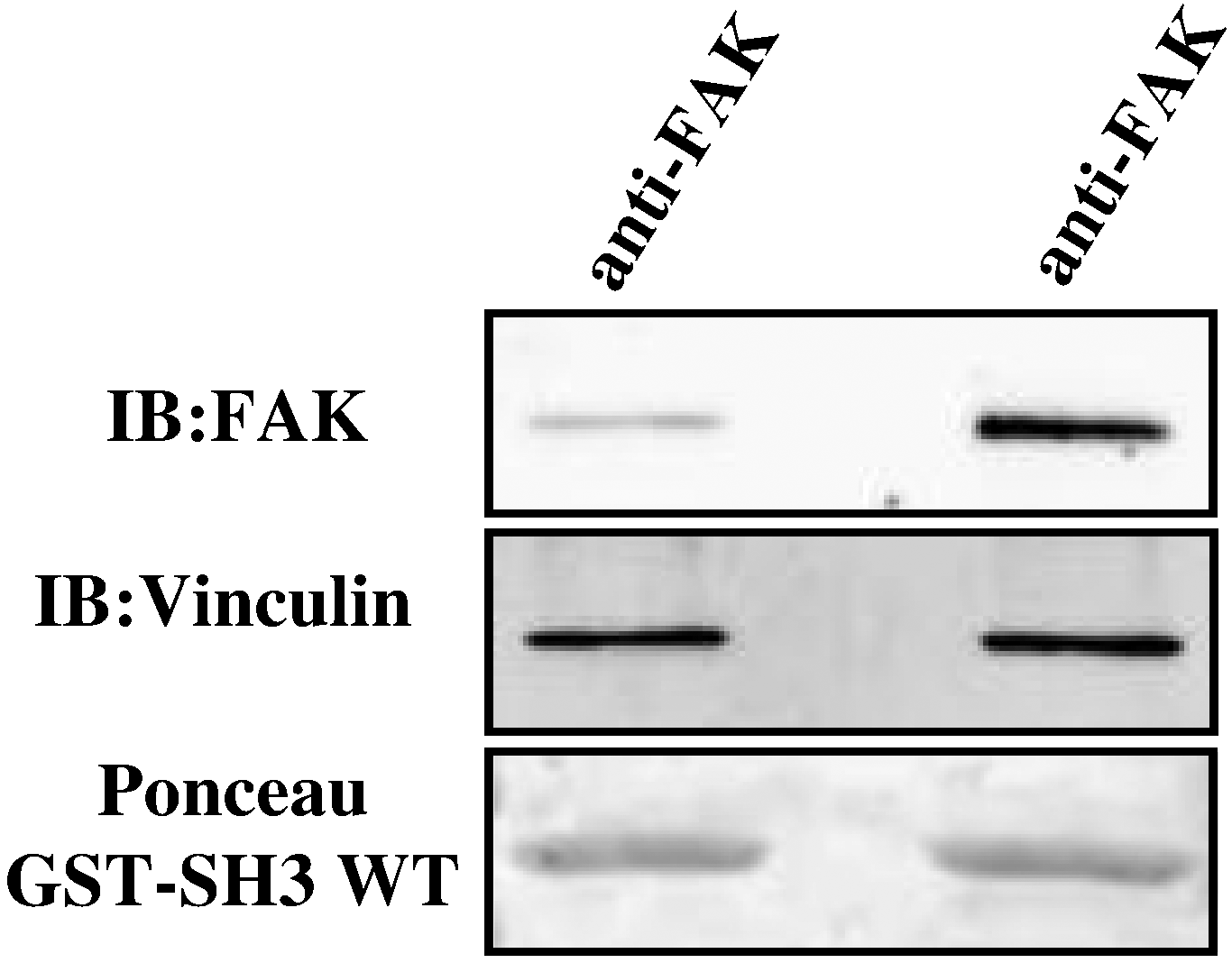

Supplement: Supplementary file 1 — GST fused WT SH3 domain was used to pull down vinculin from either whole cell or FAK depleted lysates. After immunoblotting, FAK and vinculin were detected by anti-FAK and anti-vinculin antibodies. (TIFF 170 kb) [file 18_2013_1450_MOESM1_ESM.tif]

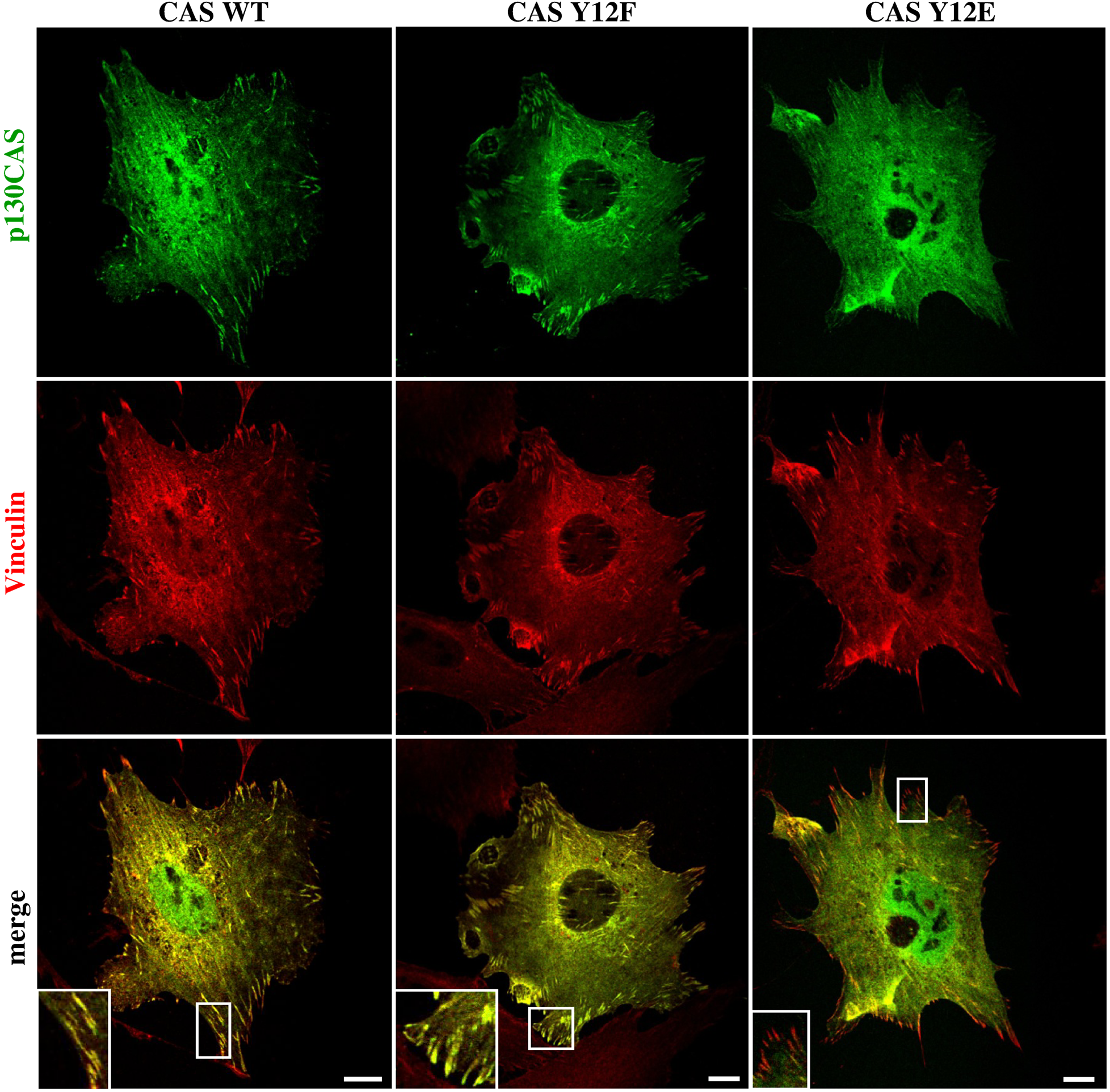

Supplement: Supplementary file 2 — CAS and vinculin co-localize in focal adhesions. MEFs expressing GFP CAS WT, Y12E and Y12F (green) were grown for 24 h on fibronectin-coated coverslips and subsequently stained for vinculin (red). CAS and vinculin localization was determined by confocal fluorescence microscopy. Scale bar: 10 μm. (TIFF 3826 kb) [file 18_2013_1450_MOESM2_ESM.tif]

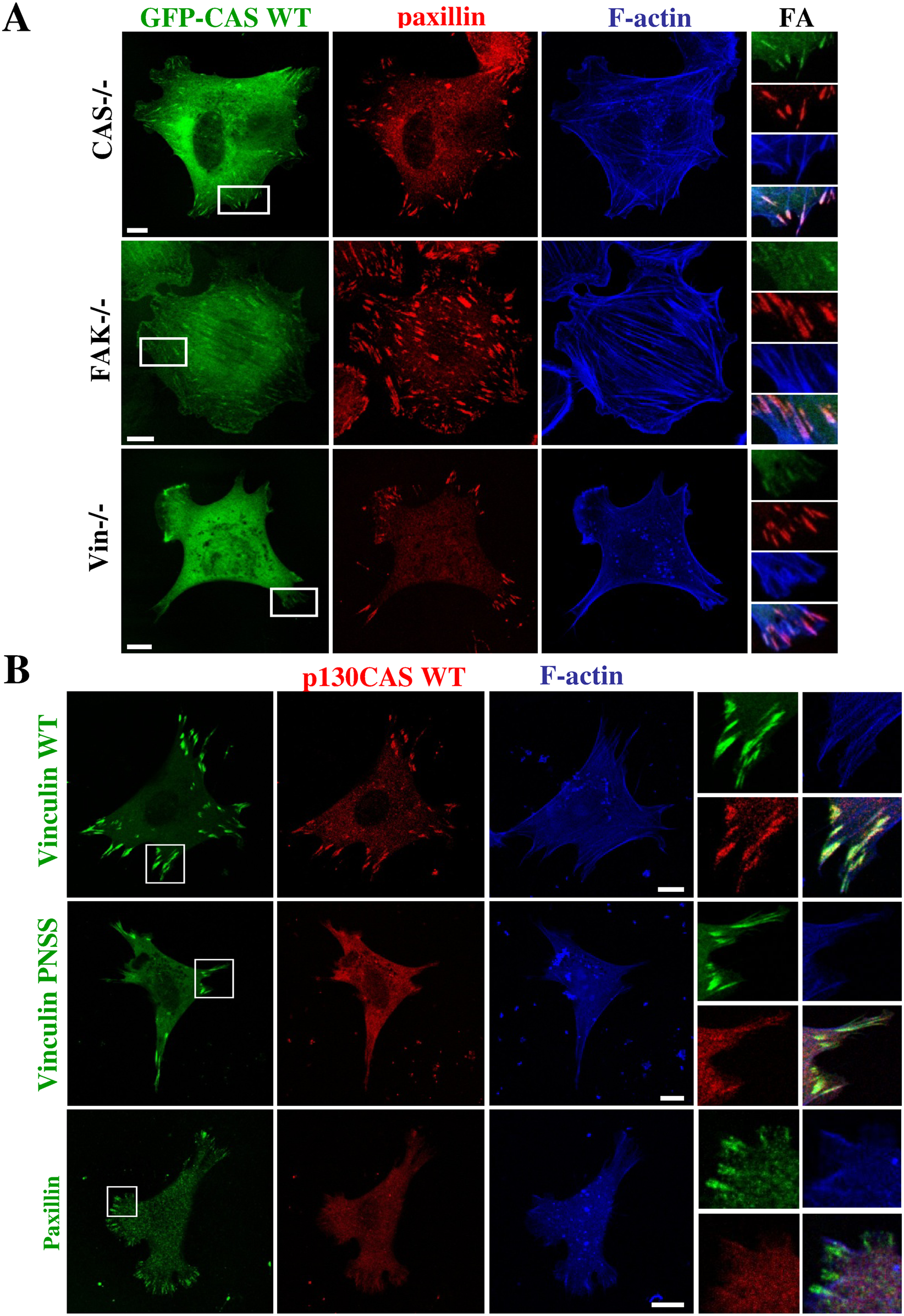

Supplement: Supplementary file 3 — Dependence of CAS localization in focal adhesions. (A) CAS−/−, FAK−/−, Vin−/− MEFs re-expressing GFP CAS WT were grown on fibronectin-coated coverslips and stained for paxillin (focal adhesion marker) and F-actin. CAS localization was determined by confocal fluorescence microscopy. Scale bar: 10 μm. (B) Vin−/− MEFs expressing mCherry CAS WT alone or with either GFP Vin WT or GFP Vin PNSS were grown on fibronectin-coated coverslips and stained for F-actin (and paxillin in case of Vin−/−). CAS localization was determined by confocal fluorescence microscopy. Scale bar: 10 μm (TIFF 3535 kb) [file 18_2013_1450_MOESM3_ESM.tif]

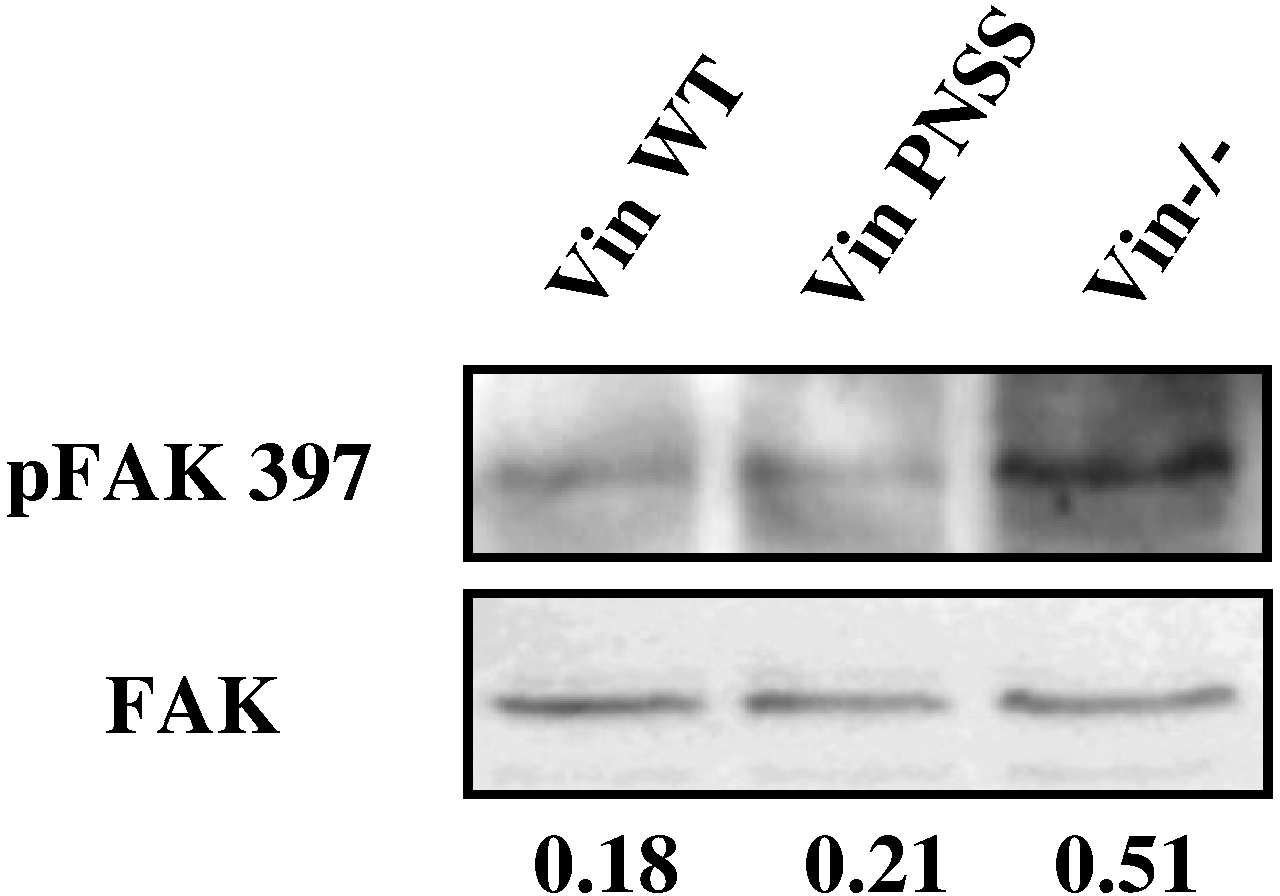

Supplement: Supplementary file 4 — Effect of vinculin on FAK phosphorylation. Vin−/− MEFs or Vin−/− MEFs re-expressing indicated vinculin variants were analyzed using a phosphospecific antibody (anti-FAK Y397) and total anti-FAK antibody. Amount of vinculin was determined by total vinculin antibody. Numbers indicate average ratios between phosphorylated and total FAK normalized to Vin WT from three independent experiments. (TIFF 139 kb) [file 18_2013_1450_MOESM4_ESM.tif]

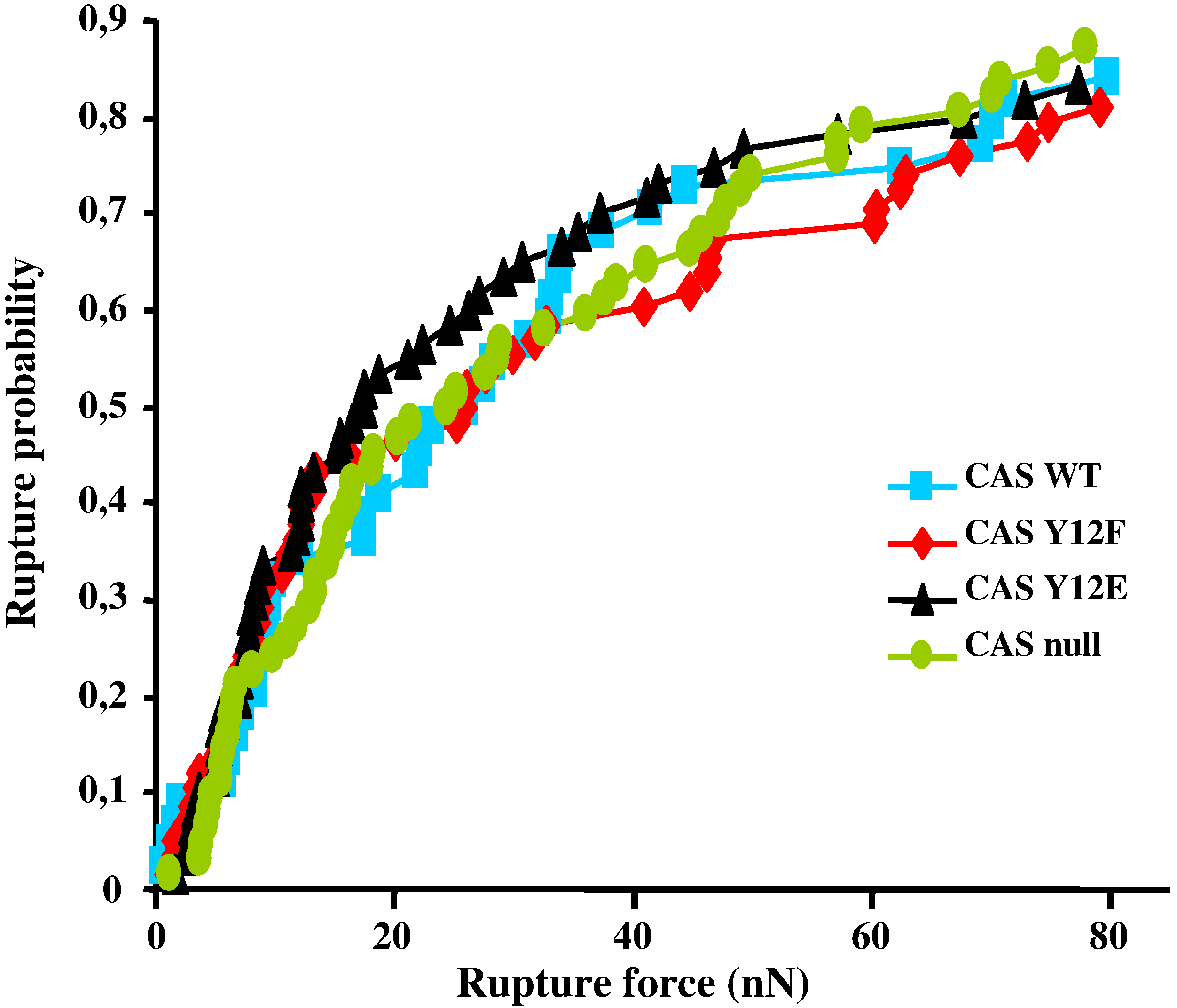

Supplement: Supplementary file 5 — Effect of CAS–vinculin interaction on adhesion strength. The curves show the dependence of fibronectin-coated bead detachment (expressed as cumulative rupture probability) on pulling force in different CAS mutant cells. (TIFF 156 kb) [file 18_2013_1450_MOESM5_ESM.tif]

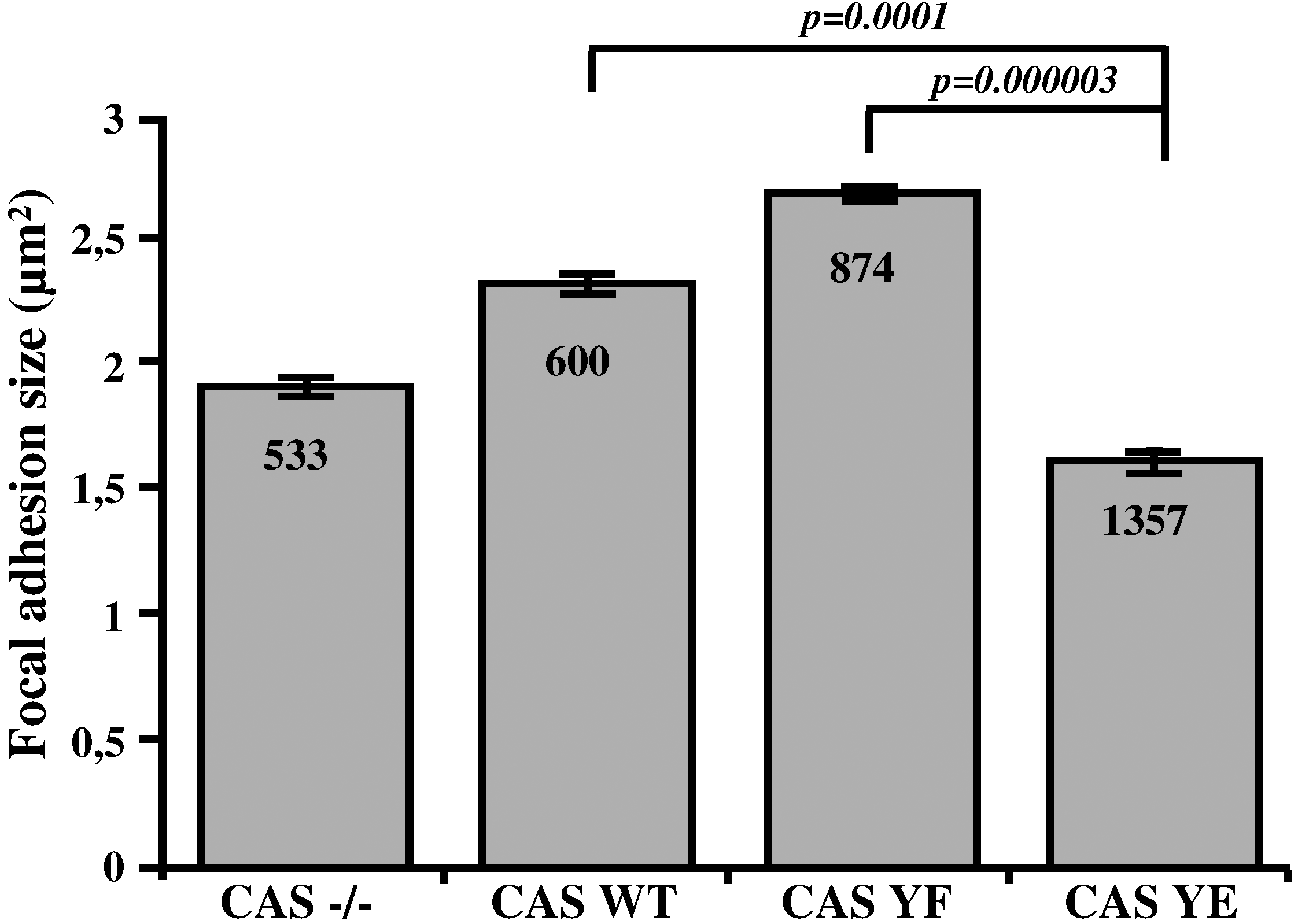

Supplement: Supplementary file 6 — Effect of tyrosine 12 phosphorylation in the CAS SH3 domain on focal adhesion size. CAS−/− MEFs re-expressing CAS Y12 variants (WT, Y12E, Y12F) were grown on fibronectin-coated coverslips and stained for paxillin (focal adhesion marker). The focal adhesion size was determined using confocal microscopy. The bar plot shows average size of adhesion structures in CAS−/− cells or cells re-expressing indicated CAS variant. Numbers in columns indicate number of analyzed focal adhesions. Error bars represent standard errors. (TIFF 240 kb) [file 18_2013_1450_MOESM6_ESM.tif]

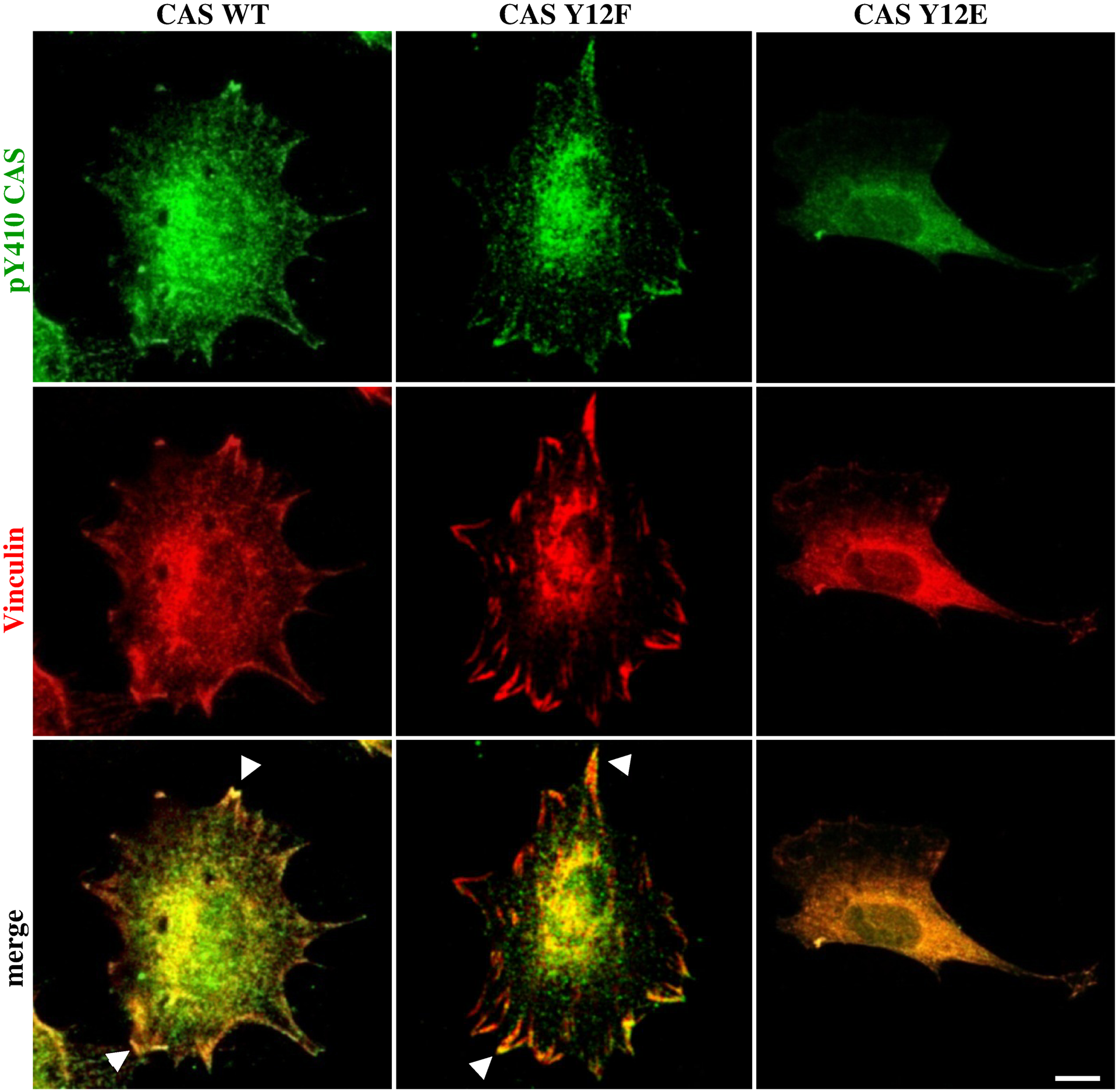

Supplement: Supplementary file 7 — Phosphorylated CAS co-localizes with vinculin in focal adhesions after stretch. CAS−/− MEFs re-expressing indicated CAS variants were seeded on fibronectin-coated PDMS stretchable membranes, incubated for 24 h, and subjected to 20 % static stretch for 10 min. Co-localization of CAS and vinculin was analyzed by confocal fluorescence microscopy. Arrows are pointing at the adhesion sites where phosphorylated CAS co-localizes with vinculin. Scale bar: 10 μm. (TIFF 2308 kb) [file 18_2013_1450_MOESM7_ESM.tif]

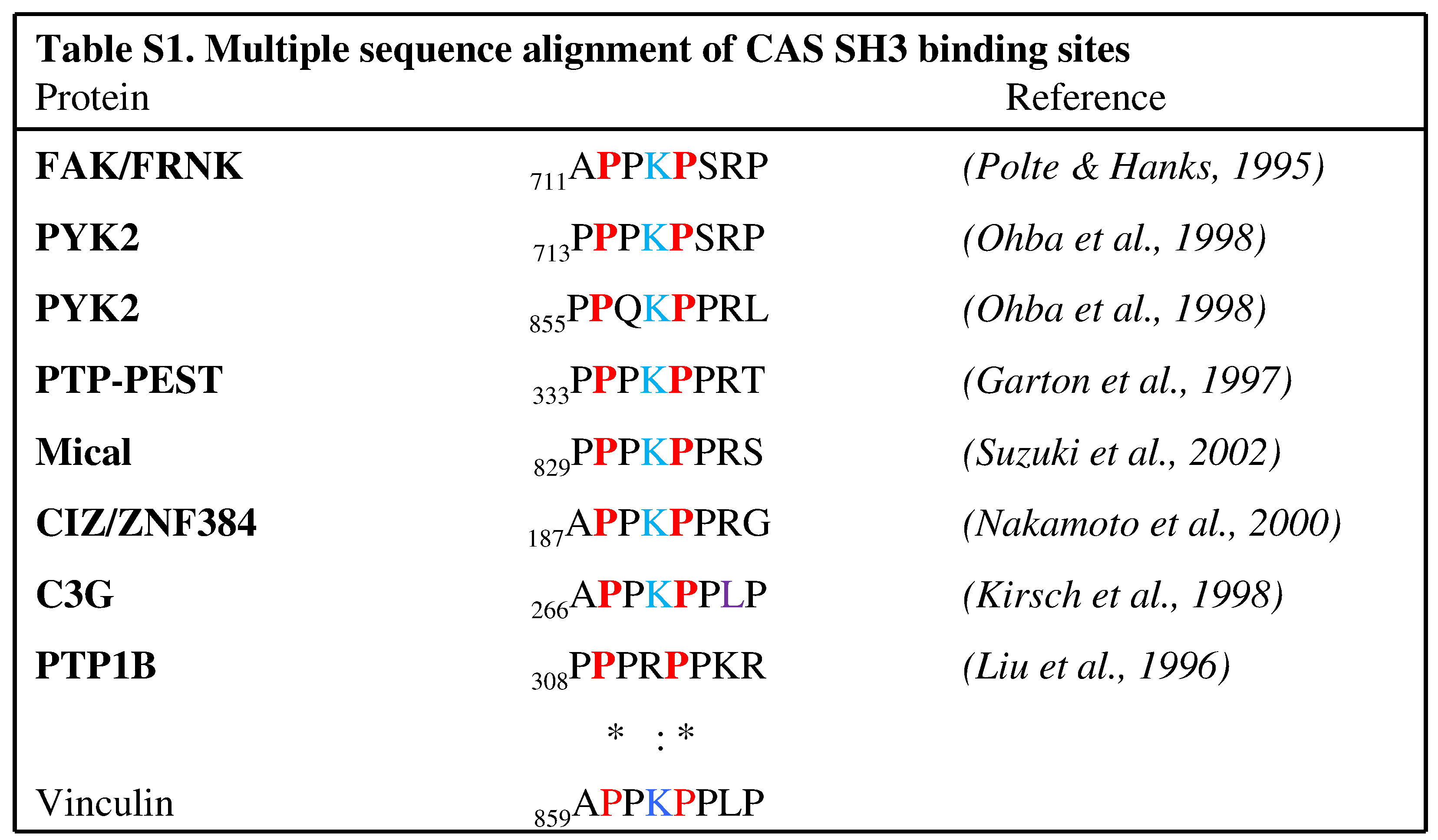

Supplement: Supplementary file 8 — Supplementary material 8 (TIFF 204 kb) [file 18_2013_1450_MOESM8_ESM.tif]
